# The brain protection of MLKL inhibitor necrosulfonamide against focal ischemia/reperfusion injury associating with blocking the nucleus and nuclear envelope translocation of MLKL and RIP3K

**DOI:** 10.3389/fphar.2023.1157054

**Published:** 2023-10-24

**Authors:** Xian-Yong Zhou, Bo Lin, Wei Chen, Rui-Qi Cao, Yi Guo, Ali Said, Taous Khan, Hui-Ling Zhang, Yong-Ming Zhu

**Affiliations:** ^1^ Jiangsu Key Laboratory of Neuropsychiatric Diseases and College of Pharmaceutical Sciences, Suzhou Key Laboratory of Drug Research for Prevention and Treatment of Hyperlipidemic Diseases, Department of Pharmacology, College of Pharmaceutical Science, Soochow University, Suzhou, Jiangsu, China; ^2^ Department of Pharmacy, COMSATS University Islamabad, Abbottabad Campus, Islamabad, Pakistan

**Keywords:** ischemic stroke, necroptosis, astrocytes, MLKL, necrosulfonamide

## Abstract

Mixed lineage kinase like protein (MLKL) is a key mediator of necroptosis. While previous studies highlighted the important role of MLKL as one of the central regulators of brain damage against acute ischemic neuronal injury, how the activation of MLKL mediates brain injuries and cell death remains unclear, especially in astrocytes. In a transient middle cerebral artery occlusion (tMCAO) rat model *in vivo*, and an oxygen-glucose deprivation and reoxygenation (OGD/Re) injury model in both primary cultured astrocytes and human astrocytes, we show that necrosulfonamide (NSA), a MLKL specific inhibitor, reduces infarction volume and improves neurological deficits in tMCAO-treated rats. In addition, NSA treatment, as well as RIP1K inhibitor Nec-1 or RIP3K inhibitor GSK-872 treatment, decreases the OGD/Re-induced leakage of LDH in both primary cultured astrocytes and human astrocytes. NSA treatment also reduces the number of propidium iodide (PI)-positive cells, and prevents the upregulation of necroptotic biomarkers such as MLKL/p-MLKL, RIP3K/p-RIP3K, and RIP1K/p-RIP1K in ischemic penumbra of cerebral cortex in tMCAO-treated rats or in OGD/Re-treated human astrocytes. Importantly, NSA treatment blocks both the nucleus and nuclear envelope localization of MLKL/p-MLKL and RIP3K/p-RIP3K in ischemic cerebral cortex induced by tMCAO. Similarly, Co-immunoprecipitation assay shows that NSA treatment decreases tMCAO- or OGD/Re- induced increased combination of MLKL and RIP3K in nuclear envelope of ischemic penumbra of cerebral cortex or of primary cultured astrocytes, respectively. RIP3K inhibitor GSK-872 also reduces tMCAO-induced increased combination of MLKL and RIP3K in nuclear envelope of ischemic penumbra of cerebral cortex. These data suggest NSA exerts protective effects against focal ischemia/reperfusion injury via inhibiting astrocytic necroptosis through preventing the upregulation of necroptotic kinases as well as blocking both the nucleus and nuclear envelope co-localization of p-MLKL and p-RIP3K. The translocation of p-MLKL, along with p-RIP3K, to the nuclear envelope and the nucleus may play a crucial role in MLKL-mediated necroptosis under ischemic conditions.

## Introduction

Stroke remains the second leading cause of death and third leading cause of disability globally (Feigin et al., 2018). Clinically, ischemic stroke accounts for 75%–80% of all stroke cases (Wang et al., 2022). To date, tissue plasminogen activator (tPA) remains the only therapeutic agent approved to treat patients with ischemic stroke (Xu et al., 2018). However, its therapeutic time window is limited to up to 4.5 h after onset of stroke symptoms (Lees et al., 2016; Li et al., 2020). Delayed tPA administration increases the risk of intracranial hemorrhage, hemorrhagic transformation, and edema (Saver et al., 2013). Therefore, finding better therapeutic targets for ischemic stroke has become an important topic.

Necroptosis is well established as a form of regulated necrotic cell death, significantly contributing to harmful events occurring with ischemic stroke. Necroptosis can be triggered by the activation of death receptors such as TNF receptor 1 (TNFR1). Once activated, TNFR1 recruits TNFR1-associated death domain (TRADD), TNFR-associated factor 2/5 (TRAF2/5), receptor-interacting kinase 1 (RIP1K), cellular inhibitors of apoptosis (cIAPs) and linear ubiquitin chain assembly complex (LUBAC) to form complex I. Once de-ubiquitinated by de-ubiquitinating enzyme CYLD or A20, RIP1K dissociates from complex I and forms complex IIa with TRADD, fas-associated protein with death domain (FADD) and caspase-8, inducing RIP1K-independent apoptosis. Under cIAPs, TAK1- or NEMO- deficient conditions, FADD, caspase-8, RIP1K and RIP3K forms complex IIb, leading to RIP1K-dependent apoptosis. When caspase-8 activation is blocked, the formation of complex IIb that includes RIP1K, RIP3K, and mixed-lineage kinase domain-like pseudokinase (MLKL) leads to necroptosis (Shan et al., 2018). The recruitment and phosphorylation of MLKL by p-RIP3K leads to MLKL oligomerization. Once oligomerized, MLKL gains the ability to translocate into plasma and multiple intracellular membranes enriched in phosphoinositides or cardiolipin, where the necrosome directly disrupts membrane integrity, resulting in necrotic death (Wang et al., 2014; Liu et al., 2020b).

As the most abundant cell type in brain, astrocytes play critical roles in normal central nervous system (CNS) physiology and in CNS injury and pathology (Barreto et al., 2011; Sims and Yew, 2017). Therefore, astrocytes are promising candidates as therapeutic targets for neuroprotection in stroke. Recently, we firstly reported that ischemic stroke leads to astrocytic necrotic cell death, showing increased expression of RIP1K, RIP3K, and MLKL. Genetic or pharmacological inhibition of RIP1K decreases infarction volume, improves neurological deficits, and attenuates astrocytic necrotic cell death (Ni et al., 2018). Necrosulfonamide (NSA) is a MLKL inhibitor targets the N-terminal domain of MLKL, and, as a result, blocks the necrotic membrane disruption mediated by MLKL (Sun et al., 2012). Zhou et al. have demonstrated that NSA exhibits neuroprotection against ischemic brain injury by degrading MLKL (Zhou et al., 2017). However, how the activation of MLKL mediates brain injuries and cell death remains largely unclear, especially in astrocytes. Here, we sought to systematically explore the effects of NSA on focal ischemia/reperfusion injury as well as its mechanism.

## Materials and methods

### Animals

Adult male Sprague-Dawley (SD) rats were purchased from The Laboratory Animal Center of Soochow University (Use license: SYXK-2016-0050; Production license: SYXK-2017-0006). All rats were housed in on-site barrier-controlled facilities with a 12 h/12 h light/dark cycle and free access to food and water. All animal procedures were approved by Soochow University Animal Care and Use Committee. All efforts were made to minimize animal suffering and to reduce the number of animals used.

### Transient middle cerebral artery occlusion model

Adult male SD rats (290-310 g) were randomly assigned to different treatment groups (n = 8 for each group) using QuickCalcs (http://www.graphpad.com/quickcalcs/). Rats were subjected to tMCAO as described previously (Qin et al., 2019; Liu et al., 2020a). Briefly, following anesthesia with 3% isoflurane, a rubber silicon-coated 4-0 monofilament suture was inserted into the right internal carotid artery until it occluded the origin of the middle cerebral artery. Reperfusion was achieved by withdrawing the suture 90 min after MCAO. Rats in sham group experienced the same operation without suture inserted. Rectal temperature was maintained at 37.0°C ± 0.5°C with a temperature-regulated heating pad during the whole procedure. NSA (Bio-Techne, 5025/10) dissolved in 10% DMSO (5 μl), GSK-872 (MedChemExpress, HY-101872) dissolved in 10% DMSO (5 μl) or vehicle, was injected intracerebroventricularly (i.c.v.) upon reperfusion, stereotaxically at the coordinates of 1.5 mm posterior to the bregma, 1.5 mm lateral from the midline, and 4.0 mm depth to the cortical surface above the lateral ventricles. Regional cerebral blood flow (rCBF) was detected using Laser-Doppler flowmetry (moorVMS; Moor Instruments, Axminster, United Kingdom). Before surgery, the laser probe was fixed to the skull (1 mm posterior to the Bregma and 5 mm lateral to the midline), rCBF was monitored during the whole tMCAO procedure. We excluded from our analysis animals that did not show a reduction of >80% in regional rCBF during MCAO and a recovery of >80% in rCBF after 10 min of reperfusion, as well as those with brain hemorrhage and those who died.

### Behavioral test

Both Neurological deficits score and cylinder test were performed as described previously (Xu et al., 2014) to evaluate various aspects of neurological function at 24 h after reperfusion.

Neurological deficits score: Rats were scored on a 6-point scale for neurological deficits. 0 represents no detectable neurological deficit; 1 represents ptosis of eyelid ipsilateral to the occluded middle cerebral artery side and/or failure to extend ipsilateral forepaw; 2 represents walk in large circles toward the ipsilateral side persistently; 3 represents walk in small circles persistently and/or rolls over repeatedly toward the ipsilateral side; 4 represents lying nearly motionless on the contralateral side; 5 represents death.

Limb-Use-Asymmetry Test (Cylinder test): Tested the forelimb-use asymmetry in a Plexiglas cylinder (HOOFAN, Wenling, Zhejiang). A total of 20 forelimb movements were recorded while rats moved vertically along the wall of the cylinder. The final score was calculated as (non-impaired forelimb movement - impaired forelimb movement)/(non-impaired forelimb movement + impaire forelimb movement + both movements).

### 2, 3, 5-Triphenyltetrazolium chloride (TTC) staining

The infarction volume was measured at 24 h after reperfusion. Each forebrain was sliced into five coronal sections of 2 mm thickness using a rat brain matrix (Harvard apparatus). Brain sections were subsequently stained with 2% TTC (T8877, Sigma), which dissolved in phosphate-buffered saline (PBS), at 37°C for 30 min, followed by fixation using 4% paraformaldehyde. ImageJ (National Institutes of Health, Bethesda, MD, United States) was used to calculate the infarction volume that was expressed as percentage of the ipsilateral volume.

### Astrocyte culture, oxygen-glucose deprivation (OGD), and cell death measurement

#### Primary cortical astrocyte culture

Primary cortical astrocytes were cultured as described previously (Qin et al., 2010; Xu et al., 2014; Du et al., 2023). Briefly, the whole cerebral cortices were isolated from postnatal day 1 SD rats. Following mechanical dissociation in PBS, cortices were digested with 2.5% trypsin at 37°C for 10 min, and then dissociated with DNase (#D7073; Beyotime, Shanghai, China). Cells were filtered through a sterile 40 μm nylon cell strainer and then cultured in DMEM/F12 (#11330, Gibco, MA, United States) containing 10% fetal bovine serum (FBS, #10099, Gibco, MA, United States) and 1% 100 U/ml penicillin/streptomycin (#C0222, Beyotime, shanghai, China). Cells were seeded on poly-L-lysine (#P1399, PLL, Sigma-Aldrich, MO, United States) coated dishes, maintained at 37 °C in a humidified atmosphere with 5% CO_2_. The medium was changed every 3 days until cells were confluent. Astrocytes were passaged at a ratio of 1:3 when the cell growth density reached 90%. The fourth passages of astrocytes were used for both cell death analysis and Western blotting analysis.

#### Human astrocytes culture

Human astrocytes (ABM, Zhenjiang, China) are cell lines isolated from human brain (cerebral cortex) and genetically engineered into immortalized cell lines. Human astrocytes were cultured in DMEM containing 10% FBS and 1% 100 U/ml penicillin/streptomycin, maintained at 37°C in a humidified atmosphere with 5% CO_2_. Human astrocytes were passaged at a ratio of 1:3 when the cell growth density reached 90%. All experiments were carried out in early passage numbers, with passage number not exceeding 20 at most.

#### Oxygen glucose deprivation/reoxygenation (OGD/Re)

Primary cortical astrocytes or human astrocytes were exposed to oxygen and glucose deprivation and reoxygenation as previously described (Zhu et al., 2021). Briefly, the medium was replaced with serum-free and glucose-free DMEM (#11966, Gibco, MA, United States). Cells were placed in a humidified and sealed hypoxic chamber (Billups-Rothenberg, CA, United States) filled with mixed gas containing 95% N_2_ and 5% CO_2_ at 37°C for an indicated time. For reoxygenation, the medium was replaced with complete DMEM/F12 (1:1) and cells were placed in normoxia at 37°C for 24 h.

NSA (0.1, 1, 10, and 100 μM), necrostatin-1 (Nec-1, Selleck, S8037, 100 μM), GSK-872 (MedChemExpress, HY-101872, 1 μM) dissolved in DMSO or vehicle was administrated upon reoxygenation. The final concentration of DMSO is 1‰.

#### Lactate dehydrogenase (LDH) leakage measurement

Cell viability was measured using LDH assay kit (Nanjing Jiancheng Bioengineering Institute, Nanjing, China). Briefly, the medium was collected after 24 h of reperfusion. Astrocytes were rinsed with PBS and lysed in 1% TritonX-100 at 37°C for 30 min. Medium and cell lysates were prepared following manufacturer’s instructions. LDH leakage was calculated as follows:

$$LDH\;leakage\;\left(\%\right)=\frac{{OD}_{medium}}{{OD}_{medium}+{OD}_{lysed\;cells}}\times 100\%$$

#### Propidium iodide (PI) staining

Propidium iodide (PI, #P4170, Sigma-Aldrich, MO, United States) was diluted in 0.9% NaCl (10 mg/ml), and was administered i.p. (15 mg/kg) to mice 1 h before sacrifice. The brains were fixed with 4% paraformaldehyde, dehydrated with saccharose, embedded in OCT (optimal cutting temperature compound, Sakura, United States), and then sliced into 16-mm thick sections in coronal plane. Subsequently, brain sections were permeabilized with 0.1% Triton X-100, blocked with 1% BSA, incubated with GFAP antibody (1:500, #53-9892-82, Thermo), and finally incubated with Hoechst (1:5,000, #33258, Sigma-Aldrich, MO, United States). Images were obtained using a confocal laser scanning microscopy (LSM 710, Carl Zeiss, Germany).

Following OGD/Re, human astrocytes were incubated with 5 μg/ml propidium iodide as well as Hoechst for 20 min. Then images were obtained using fluorescence microscope (IX73, Olympus, Japan).

### Nuclear and cytoplasmic extraction

Nuclear and cytoplasmic extraction was performed using NE-PER Nuclear and Cytoplasmic Extraction Reagents (#78833, Thermo) following manufacturer’s instructions. Briefly, tissue from peri-infarct region was cut into small pieces and then homogenized using a tissue grinder in the appropriate volume of CER I. The tubes were incubated on ice for 10 min, followed by the addition of CER II. The tubes were incubated on ice for 1 min and then centrifuged at maximum speed for 5 min. The supernatant (cytoplasmic extract) was transferred to clean tubes. Next, the insoluble fraction was suspended in NER and vortex for 15 s every 10 min, for a total of 40 min, followed by centrifugation at maximum speed for 10 min. The supernatant fraction (nuclear extract) was transferred to clean tubes and stored at −80 °C.

### Nuclear envelope protein extraction

Nuclear envelope protein extraction was performed using Minute Nuclear Envelope Protein Extraction Kit (NE-013, Invent Biotechnologies) following manufacturer’s instructions. After OGD/Re, human astrocytes or tissue from peri-infarct region were collected by low speed centrifugation. The supernatant was removed completely and the pellet was incubated with buffer A for 10 min. Following centrifugation at 14,000 × g for 30 s, the filter was discarded and the supernatant was removed. Then the tubes were incubated with buffer B for 10 min and then centrifuged at 5000 × g for 5 min at 4°C. The supernatant was transferred to new tubes. Following the addition of PBS, the tubes were inverted 10 times and then centrifuged at 16,000 × g at 4°C for 15 min. The supernatant was removed and the pellet was stored.

### Co-immunoprecipitation

Co-immunoprecipitation was performed using Protein A/G Magnetic Beads (MedChemExpress). Briefly, anti-MLKL antibody was added to the beads and incubated with rotation for 30 min at room temperature. Human astrocytes whole-cell extracts were added to the beads-antibody complex and incubated with rotation for 30 min at room temperature. Followed by the addition of SDS-PAGE Loading Buffer, samples were heated at 95°C for 10 min. Then, magnetic separation was performed and the supernatant was collected for SDS-PAGE.

#### Western blotting assay

Proteins were extracted from astrocyte lysate using lysis buffer (10 mM Tris HCl, 150 mM NaCl, 1% TritonX-100, 1% sodium deoxycholate, 0.1% sodium dodecyl sulfate, 5 mM EDTA; pH 7.4) with protease inhibitor and phosphatase inhibitor. Protein samples were normalized using Pierce BCA Protein Assay Kit (#23227, Thermo), followed by heating at 95°C for 10 min. PVDF membranes were used for protein transfer and blocked in 5% BSA at room temperature for 1 h. Membranes were incubated with specified primary antibodies (Supplementary Table S1) at 4°C overnight, followed by incubation with corresponding secondary antibodies (Supplementary Table S1) at room temperature for 1 h. Odyssey scanner (LI-COR Biosciences, NE, United States) was used to image. Densitometry analyses of bands were performed using ImageJ.

### Statistical analysis

All statistical analyses were conducted using GraphPad Prism. All error bars represent mean ± SD. One-way or Two-way analysis of variance (ANOVA) was conducted to measure statistical significance. Significance was defined as *p* < 0.05.

## Results

### NSA produces neuroprotection against I/R-induced acute brain injury

**FIGURE 1:**
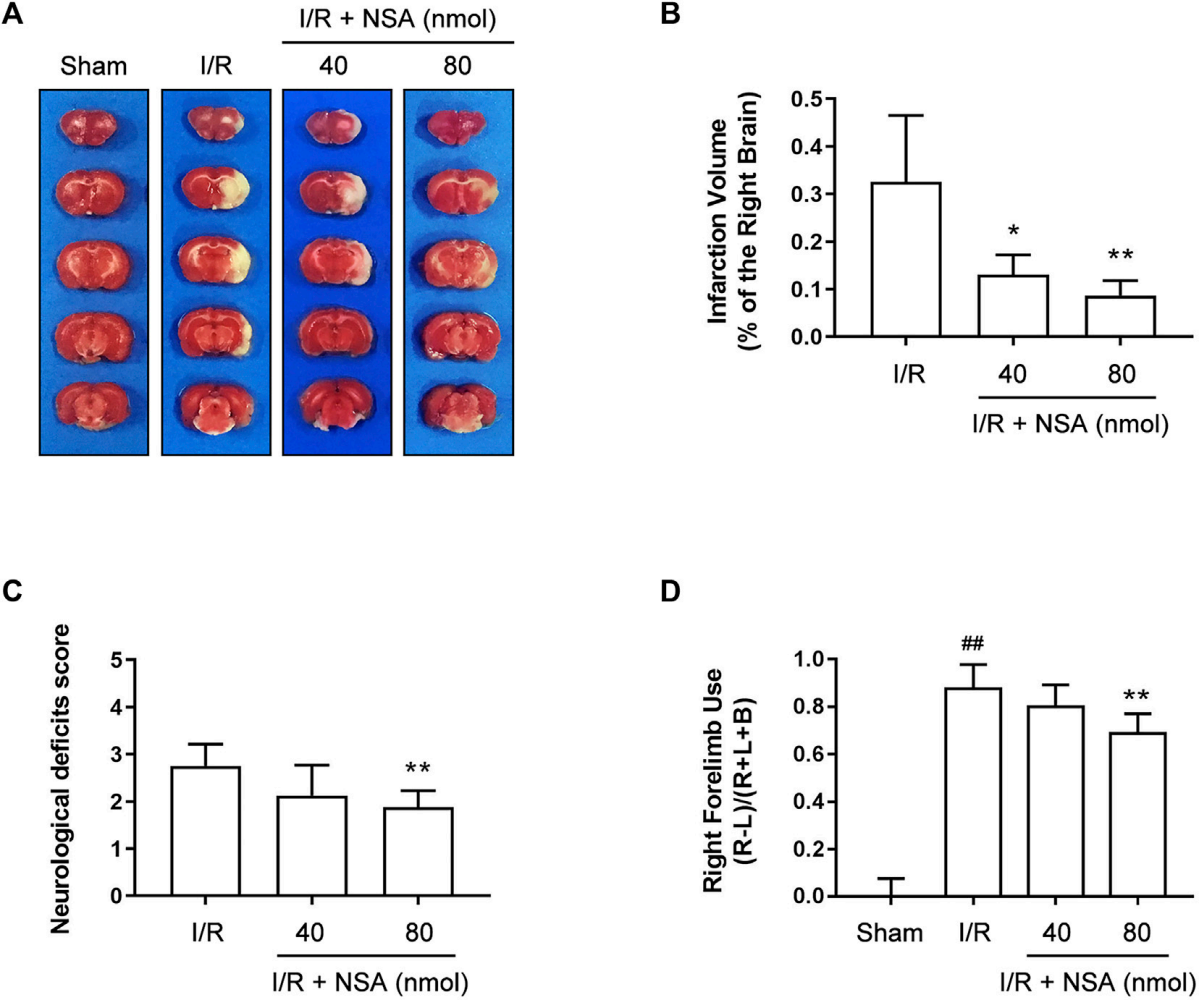
NSA decreases cerebral infarction volume and improves neurological functions at 24 h after reperfusion. **(A)** Representative photographs of rats’ brain slices stained with TTC. **(B)** Quantitative analysis of infarction volume. Neurological deficits score **(C)** and cylinder test **(D)** were performed to evaluate various aspects of neurological function at 24 h after reperfusion. N = 8 biological replicates. Data shown are the mean with error bars representing ±SD. ##*p* < 0.01 vs. sham group; *p < 0.05, **p < 0.01 vs. I/R group.

The protective effect of NSA against I/R-induced brain injury has been reported previously in mice (Zhou et al., 2017) but not in rats. To validate the neuroprotection of NSA against I/R-induced acute brain injury in rats, different doses of NSA (40 nmol, 80 nmol) was administrated intracerebroventricularly upon reoxygenation. As shown in Figures 1A, B, NSA at both 40 nmol and 80 nmol significantly decreased cerebral infarction volume compared to I/R group. Consistent with the reducing infarction volume, NSA at 80 nmol greatly decreased Neurological deficits score (Figure 1C) as well as right forelimb use (Figure 1D). Collectively, these data suggest that NSA produces neuroprotective effects against I/R-induced acute brain injury in rats. 80 nmol was chosen as the optimum dose of NSA for further experiments *in vivo*.

### NSA protects both primary astrocytes and human astrocytes against OGD/Re-induced injury

**FIGURE 2:**
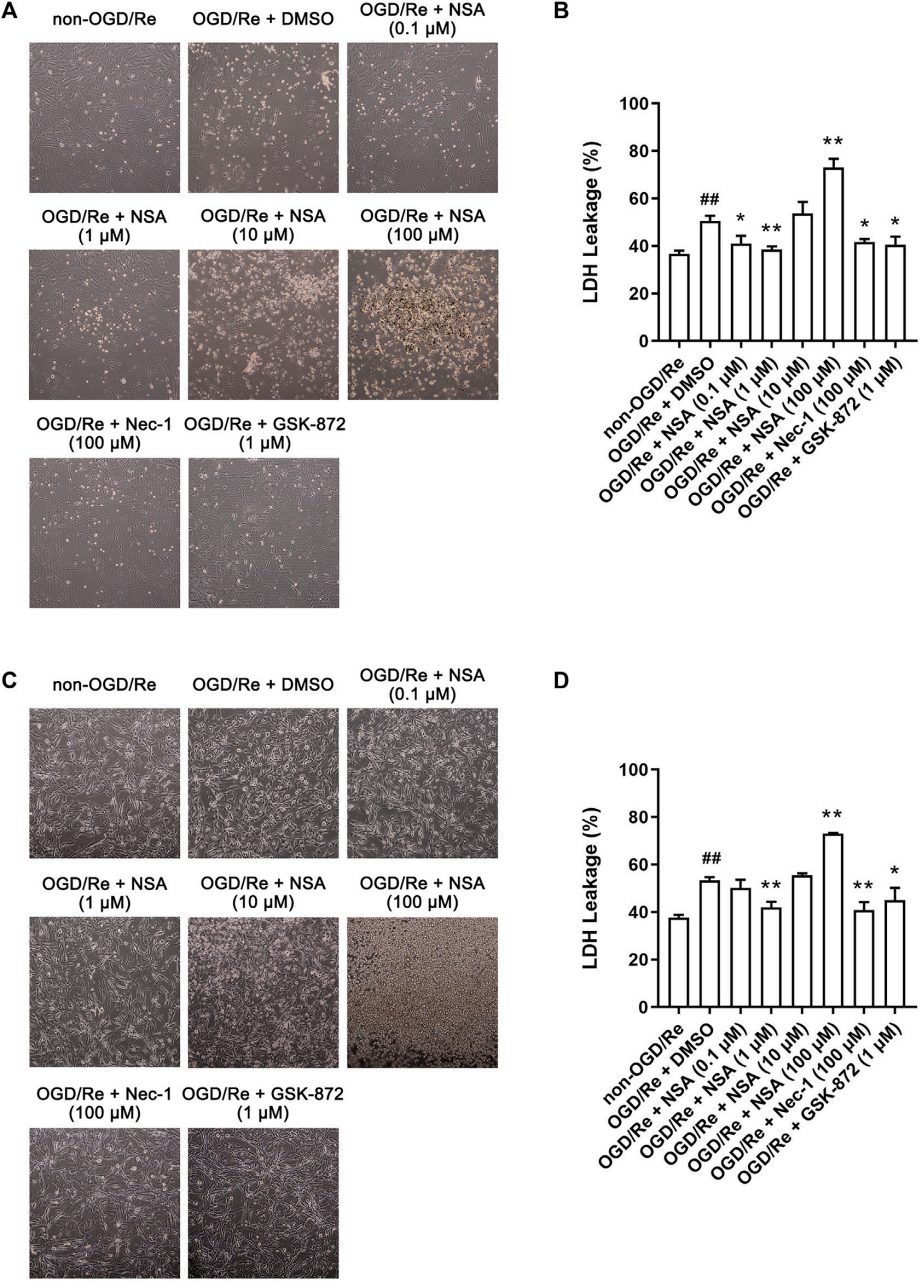
NSA protects primary cultured astrocytes and human astrocytes against OGD/Re-induced cell injury. Representative light microscopy images of **(A)** primary astrocytes and **(C)** human astrocytes exposed to OGD for 6 h or 3 h, respectively, followed by reoxygenation for 24 h. Primary astrocytes and human astrocytes were treated with different concentrations of NSA, Nec-1, GSK-872 or vehicle upon reoxygenation. **(B)** Columns represent quantitative analysis of LDH leakage in panel **(A)**. **(D)** Columns represent quantitative analysis of LDH leakage in panel **(C)**. N = 3 biological replicates. Data shown are the mean with error bars representing ±SD. ^##^ *p* < 0.01 vs. non-OGD/Re group, *p < 0.05, **p < 0.01 vs. OGD/Re + DMSO group.

To further investigate whether NSA attenuates astrocytic cell death, we established OGD/Re model *in vitro* to mimic I/R injury. RIP1K inhibitor Nec-1 and RIP3K inhibitor GSK-872 were used as positive control. LDH leakage assay in primary astrocytes showed that not only Nec-1 (100 μM) and GSK-872 (1 μM), but also NSA at 0.1 μM and 1 μM decreased the leakage of LDH and increased the number of astrocytes (Figures 2A, B). NSA at 1 μM showed the strongest protective effect whereas NSA at 10 μM and 100 μM is toxic to primary astrocytes. Then we re-performed this experiment in human astrocytes. Similarly, LDH leakage results showed that NSA at 1 μM, as well as Nec-1 (100 μM) and GSK-872 (1 μM), protected human astrocytes against OGD/Re-induced injury (Figures 2C, D). NSA at 100 μM is toxic to human astrocytes. Therefore, 1 μM was chosen as the optimum concentration of NSA for further experiments *in vitro*.

### NSA inhibits ischemia-induced necroptotic cell death

**FIGURE 3:**
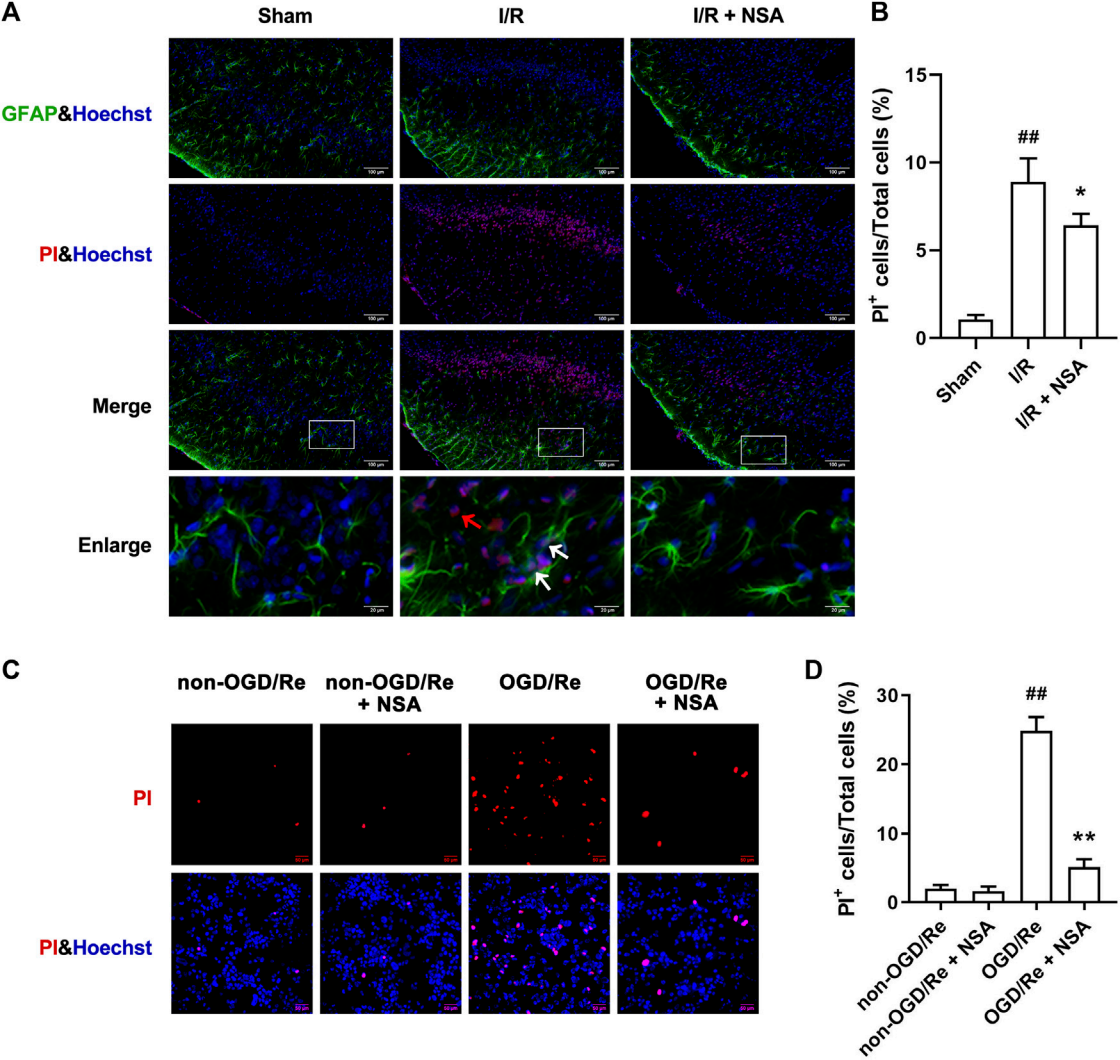
NSA reduces the number of PI-positive cells. **(A)** Cell death was assessed with PI, GFAP, and Hoechst staining (PI: red; GFAP: green; Hoechst: blue). White arrows represent PI^+^GFAP^+^ cells. Red arrows represent PI^+^GFAP^−^ cells. **(B)** Columns represent quantitative analysis in panel **(A)**. N = 3 biological replicates. Data shown are the mean with error bars representing ±SD. ^##^ *p* < 0.01 vs. Sham group, *p < 0.05 vs. I/R group. **(C)** Human astrocytes were exposed to OGD for 3 h followed by reoxygenation for 24 h. Cell death was assessed with PI and Hoechst staining (PI: red; Hoechst: blue). **(D)** Columns represent quantitative analysis in panel **(C)**. N = 3 biological replicates. Data shown are the mean with error bars representing ±SD. ^##^ *p* < 0.01 vs. non-OGD/Re group, **p < 0.01 vs. OGD/Re group.

**FIGURE 4:**
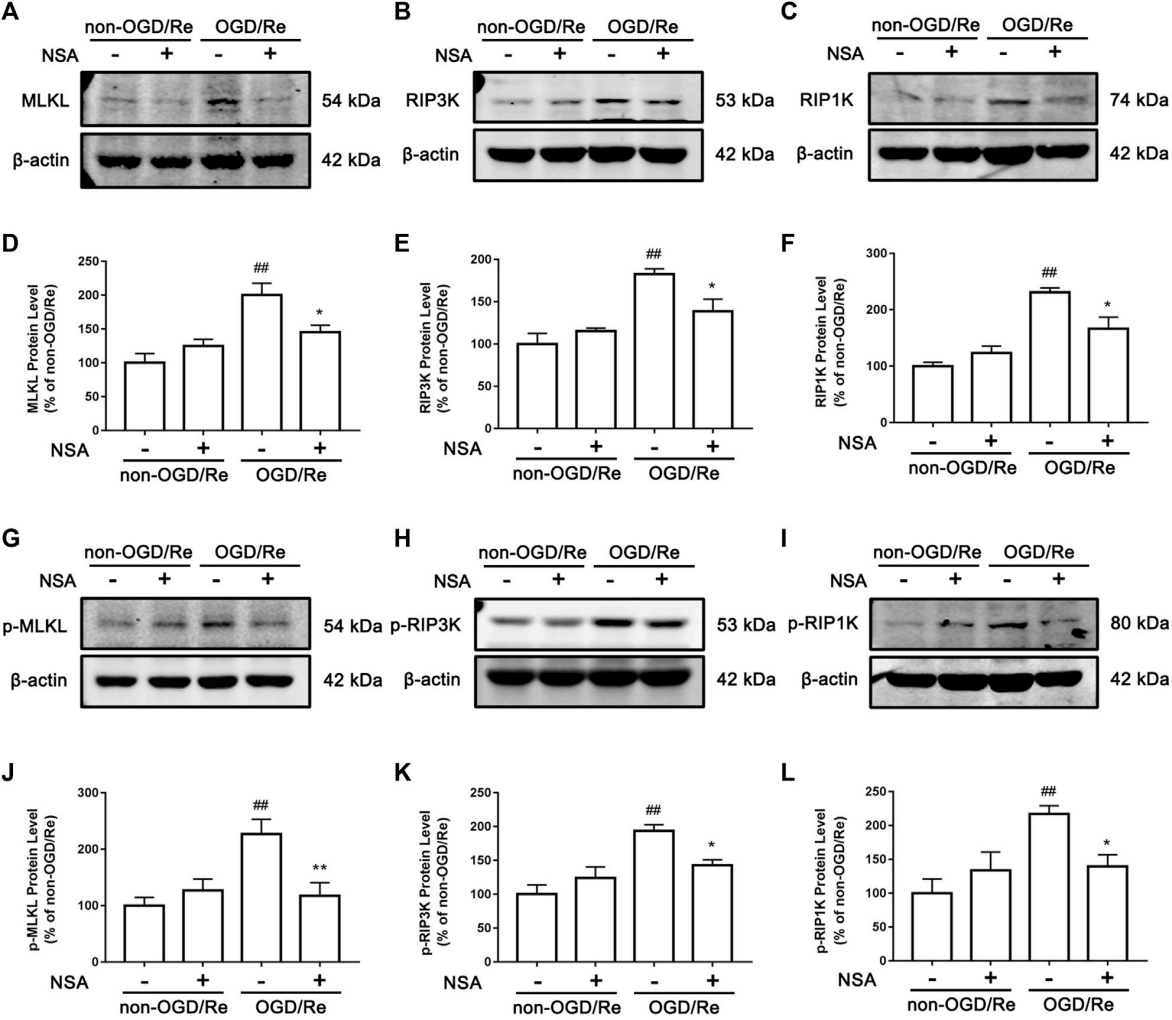
NSA inhibits the levels of necroptosis-related proteins. Primary cultured astrocytes from rats were exposed to OGD for 6 h followed by reoxygenation for 24 h. Western blotting analysis of the protein levels of MLKL **(A)**, RIP3K **(B)**, RIP1K **(C)**, p-MLKL **(G)**, p-RIP3K **(H)**, and p-RIP1K **(I)**. **(D–F)** Columns represent quantitative analysis of MLKL, RIP3K, and RIP1K. **(J–L)** Columns represent quantitative analysis of p-MLKL, p-RIP3K, and p-RIP1K. β-actin protein was used as a loading control. N = 3 biological replicates. Data shown are the mean with error bars representing ±SD. ^##^ *p* < 0.01 vs. non-OGD/Re group; **p < 0.01, *p < 0.05 vs. OGD/Re group.

Next, we determined if NSA exerts protective effects by inhibiting ischemia-induced necroptotic death of astrocytes. We observed an increase in the number of PI-positive cells in of ischemic penumbra of cerebral cortex, while NSA treatment significantly reduced the number of PI-positive cells (Figures 3A, B). Meanwhile, NSA treatment also greatly reduced the number of OGD/Re-induced PI-positive human astrocytes (Figures 3C, D). Moreover, NSA prevented the upregulation of necroptotic kinases including MLKL/p-MLKL, RIP3K/p-RIP3K, and RIP1K/p-RIP1K in OGD/Re-treated astrocytes (Figure 4). These findings suggest that NSA inhibits ischemia-induced necroptotic cell death.

### NSA blocks I/R-induced nuclear localization as well as nuclear envelope localization of MLKL and RIP3K

**FIGURE 5:**
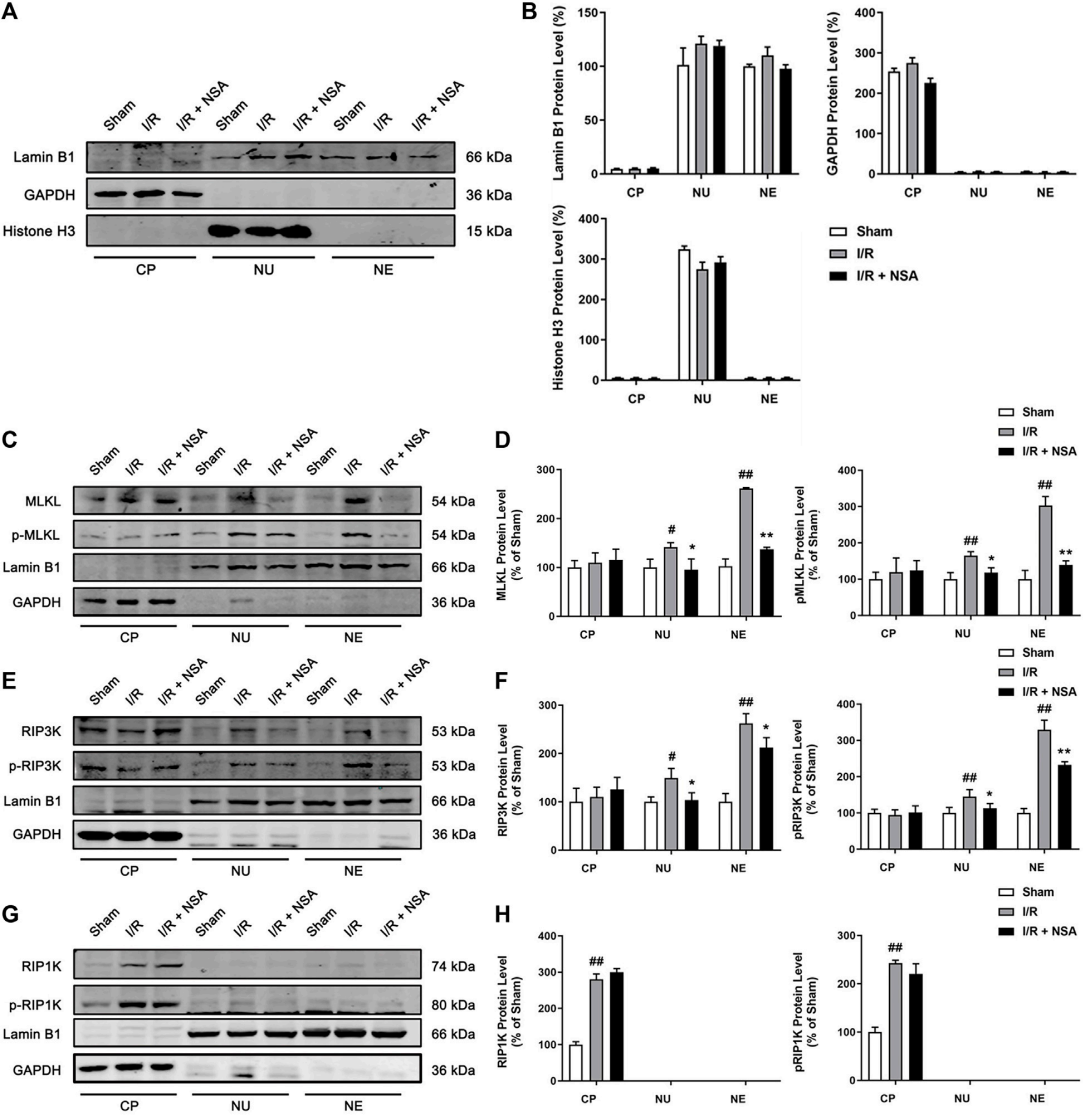
NSA blocks the nucleus and nuclear envelope localization of MLKL/p-MLKL, and RIP3K/p-RIP3K. **(A, B)** The identification for the extraction of cytoplasmic, nuclear and nuclear envelope proteins. Lamin B1, GAPDH and Histone H3 represents nuclear envelope, cytoplasmic and nuclear marker, respectively. Representative Western blotting images for the protein levels of **(C)** MLKL/p-MLKL, **(E)** RIP3K/p-RIP3K, and **(G)** RIP1K/p-RIP1K in the peri-infarct region at 24 h after reperfusion following cerebral ischemia for 90 min in rats. **(D, F, H)** Quantitative analysis of the protein levels in panel C, E and G, respectively. N = 3 biological replicates. Data shown are the mean with error bars representing ±SD. ^##^ *p* < 0.01, ^#^ *p* < 0.05 vs. sham group; **p < 0.01, *p < 0.05 vs. I/R group. (CP: cytoplasm, NU: nucleus, NE: nuclear envelope).

MLKL is known to translocate into plasma membrane and multiple intracellular membranes enriched in phosphoinositides or cardiolipin (Dondelinger et al., 2014; Wang et al., 2014). Phosphoinositides are also present in nuclear envelope (Larijani and Poccia, 2009), raising the possibility that phosphoinositides might also draw MLKL to nuclear envelope. It is also reported that MLKL could translocate into the nucleus along with RIP1K and RIP3K before necroptotic cell death induced by TNF + BV6+z-VAD.fmk in both HT29 cells and mouse embryonic fibroblasts (Yoon et al., 2016). To further investigate whether MLKL translocates to nucleus or nuclear envelope upon ischemic stroke, nuclear and cytoplasmic extraction as well as nuclear envelope protein extraction was performed. The Western blotting results showed that nuclear, cytoplasmic, and nuclear envelope protein were successfully extracted (Figure 5A). As shown in Figures 5C–F, MLKL/p-MLKL, and RIP3K/p-RIP3K were located in both nucleus and nuclear envelope in the peri-infarct region at 24 h after reperfusion. In the presence of NSA, the protein levels of MLKL/p-MLKL and RIP3K/p-RIP3K in both nucleus and nuclear envelope were downregulated (Figures 5C–F), indicating that the nucleus and nuclear envelope translocation of RIP3K depend on MLKL. Surprisingly, we did not find the localization of RIP1K and p-RIP1K in either nucleus or nuclear envelope as our expected (Figures 5G, H). These results indicate that NSA blocks I/R-induced nucleus and nuclear envelope translocation of MLKL and RIP3K.

### NSA reduces ischemia-induced combination of MLKL and RIP3K in nuclear envelope

**FIGURE 6:**
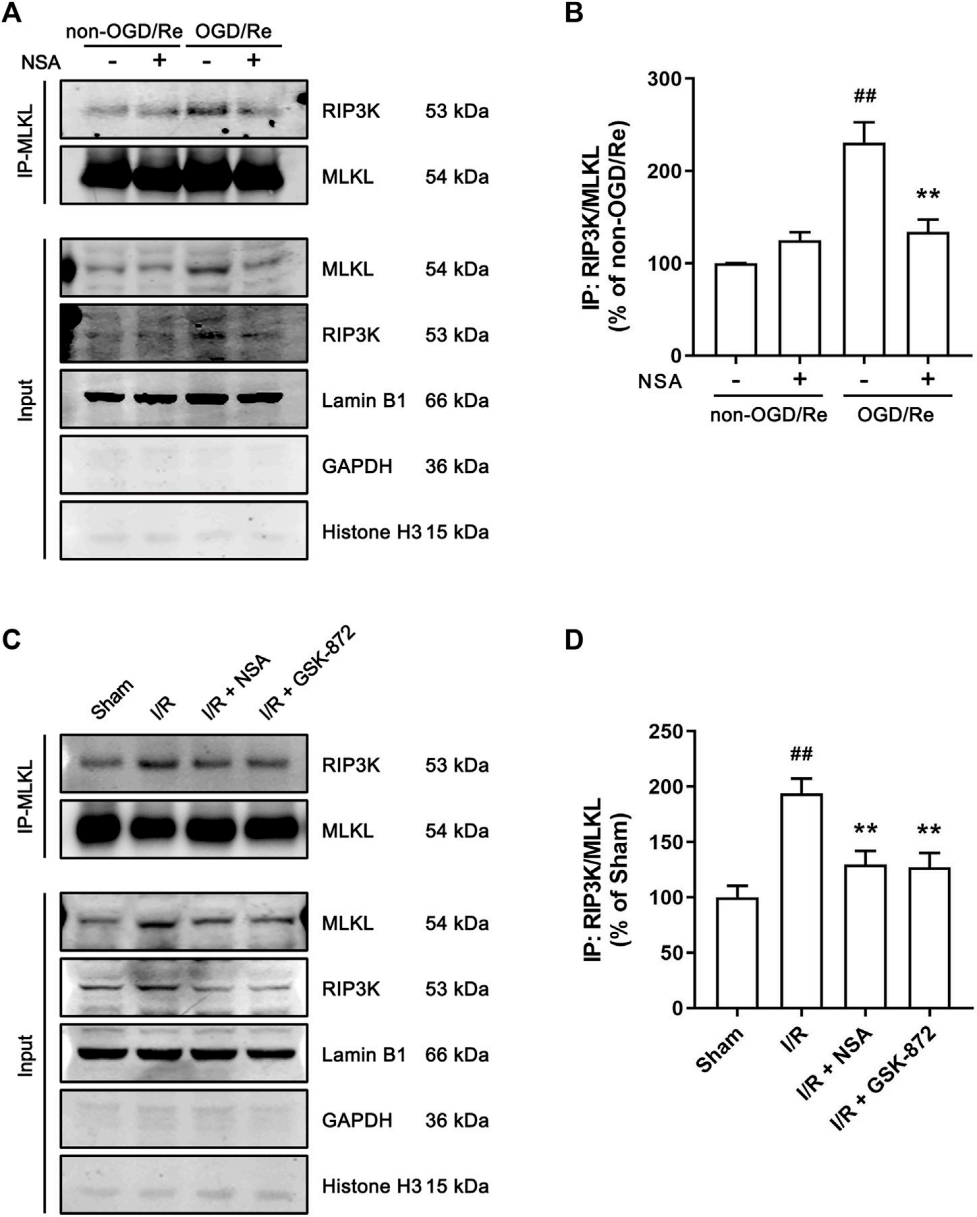
NSA reduces the combination of MLKL and RIP3K in nuclear envelope in both human astrocytes and ischemic penumbra of cerebral cortex. **(A)** Human astrocytes were exposed to OGD for 3 h followed by reoxygenation for 24 h. Co-immunoprecipitation of RIP3K with MLKL from the nuclear envelope extracts. Lamin B1, GAPDH and Histone H3 represents nuclear envelope, cytoplasmic and nuclear marker, respectively. **(B)** Columns represent quantitative analysis of immunoblots in panel **(A)**. N = 3 biological replicates. Data shown are the mean with error bars representing ±SD. ^##^ *p* < 0.01 vs. non-OGD/Re group; **p < 0.01 vs. OGD/Re group. **(C)** Mice subjected to tMCAO were treated with NSA or GSK-872. Co-immunoprecipitation of RIP3K with MLKL from the nuclear envelope extracts. **(D)** Columns represent quantitative analysis of immunoblots in **(C)**. N = 3 biological replicates. Data shown are the mean with error bars representing ±SD. ^##^ *p* < 0.01 vs. Sham group; **p < 0.01 vs. I/R group.

Since the translocation of RIP3K depends on the function of MLKL, we next examined whether MLKL and RIP3K co-locate to nuclear envelope as a complex. As shown in Figure 6, the interaction between MLKL and RIP3K was largely enhanced in nuclear envelope in human astrocytes subjected to OGD/Re (Figures 6A, B) and in ischemic penumbra of cerebral cortex (Figures 6C, D), while NSA treatment significantly inhibited the combination of MLKL and RIP3K in nuclear envelope (Figures 6A–D). Besides, GSK-872 also reduced the combination of MLKL and RIP3K in ischemic penumbra of cerebral cortex (Figures 6C, D), which showed that the nuclear envelope-localized MLKL-RIP3K complex was dependent on activity of RIP3K and MLKL.

## Discussion

In this study, we explored the effects of MLKL inhibitor NSA on ischemic stroke as well as its potential mechanisms. We validated that NSA produces neuroprotective effects against ischemia/reperfusion-induced brain injury, and NSA, as well as RIP1K inhibitor Nec-1 and RIP3K inhibitor GSK-872, decreased OGD/Re-induced injury in human astrocytes. Besides, we found that NSA reduces the number of tMCAO- or OGD/Re-induced PI-positive cells, and prevents the upregulation of necroptotic key kinases MLKL/p-MLKL, RIP3K/p-RIP3K, and RIP1K/p-RIP1K in OGD/Re-treated astrocytes. Most importantly, NSA blocks the both nucleus and nuclear envelope localization of MLKL/p-MLKL and RIP3K/p-RIP3K in the peri-infarct region at 24 h after reperfusion. We further showed that NSA reduces the combination of MLKL and RIP3K in nuclear envelope in human astrocytes subjected to OGD/Re and in ischemic penumbra of cerebral cortex. GSK-872 was also found to reduce the combination of MLKL and RIP3K in ischemic penumbra of cerebral cortex. These findings help us to understand the pharmacology of NSA in treatment of ischemic stroke and promote the development of new strategy against ischemic-induced brain injury.

MLKL has been identified as a key executor of necroptosis. When necroptosis occurs, RIP3K undergoes auto-phosphorylation, inducing the recruitment and phosphorylation of MLKL. The phosphorylation of MLKL is the cue for its oligomerisation and translocation. This translocation is facilitated by N-terminal domain of MLKL, which possesses a patch of positively charged amino acid that enables MLKL to interact with phospholipids such as phosphoinositides and cardiolipin. In this way, oligomeric MLKL moves to plasma membrane and multiple intracellular membranes enriched in phosphoinositides or cardiolipin, disrupts membrane integrity and finally causes cells to undergo necrotic cell death.

The effects of MLKL inhibitor NSA are controversial. The earliest studies established that NSA targets the Cys86 residue of MLKL and thus targets human MLKL, but not mouse MLKL (Sun et al., 2012). However, recently emerging evidence from rodent models confirmed the inhibitory effects of NSA on necroptosis, therefore improving neurological function after ischemic brain injury (Zhou et al., 2017) as well as alleviating spinal cord damage (Jiao et al., 2020). Here, we present a detailed assessment of the neuroprotective effects of MLKL against ischemia/reperfusion-induced injury in rats both *in vivo* and *in vitro*.

It is reported that inhibition of MLKL through siRNA diminished RIP1K-RIP3K-MLKL interaction and attenuated neuronal death induced by OGD/zVAD (Qu et al., 2016). We determined if NSA exerts important protective effects by reducing the levels of necroptosis-related proteins in astrocytes. Interestingly, NSA treatment not only reduced the levels of MLKL in astrocytes after OGD/Re injury, but also reduced the levels of p-MLKL, RIP3K/p-RIP3K, and RIP1K/p-RIP1K. Since NSA targets the N-terminal domain of MLKL without interfering with phosphorylation by RIP3K, it could be suspected that ischemia/reperfusion induces a positive feedback between MLKL and its upstream signals RIP1K and RIP3K. It is reported that activated MLKL reversely enhance activation of RIP1K and RIP3K and expressional upregulation of CYLD via improvement of intracellular ROS (Ding et al., 2019). The specific mechanism is still to be studied.

The important findings of the current study are that we firstly provide evidences that the increased complex of p-MLKL and p-RIP3K is translocated to nucleus and nuclear envelope in both ischemic penumbra of cerebral cortex and ischemic astrocytes, while no nucleus and nuclear envelope translocations of p-RIP1K were seen. We have not yet investigated MLKL translocation to plasma membrane and other intracellular membrane, thus we are unable to draw any conclusions on how common the nuclear translocation is. The nuclear translocation of MLKL might facilitate necroptosis (Yoon et al., 2016; Weber et al., 2018), which might help to explain the positive feedback between MLKL and its upstream signals RIP1K and RIP3K. The expression of the MLKL T357A/S358A mutant in HT29 cells blocked TNF + BV6+z-VAD.fmk-induced nuclear translocation of RIP1K and RIP3K (Yoon et al., 2016), suggesting that the translocation of these two protein kinases depends on that of MLKL, and that the association of MLKL with the cell membrane in necroptotic death is preceded by the translocation of phosphorylated MLKL, along with RIP1K and RIP3K, to the nucleus. In this study, we revealed that NSA downregulates ischemia/reperfusion-induced increases of MLKL/p-MLKL, and RIP3K/p-RIP3K in both nucleus and nuclear envelope, and reduces OGD/Re-induced combination of MLKL and RIP3K in nuclear envelope of astrocytes, indicating that the translocation of p-MLKL, along with p-RIP3K, to the nuclear envelope and the nucleus may play a crucial role in MLKL-mediated necroptosis under ischemic conditions. Although we cannot rule out that the nuclear envelope complex of MLKL and RIP3K exerts independently on their nuclear translocation, we speculate that the nuclear envelope co-localization of MLKL and RIP3K is only a process for their nuclear translation from the cytoplasm.

In addition, RIP1K and p-RIP1K have no obvious nuclear localization or nuclear envelope localization in the peri-infarct region at 24 h after reperfusion following cerebral ischemic for 90 min. This is in accordance with a previous study by Yin et al. that RIP3K was upregulated and translocated to the nucleus while RIP1K was not affected in hippocampal CA1 neurons after global cerebral ischemia/reperfusion injury (Yin et al., 2015). But this investigation by Yin et al. did not detect the MLKL level in the neuronal nucleus. Our present data and Yin et al.‘s different from the findings of Yoon et al.‘s showing the translocation of RIP1K to the nucleus (Yoon et al., 2016). The differences may be due to the differences in experimental subjects, methods, stimuli and so on.

**FIGURE 7:**
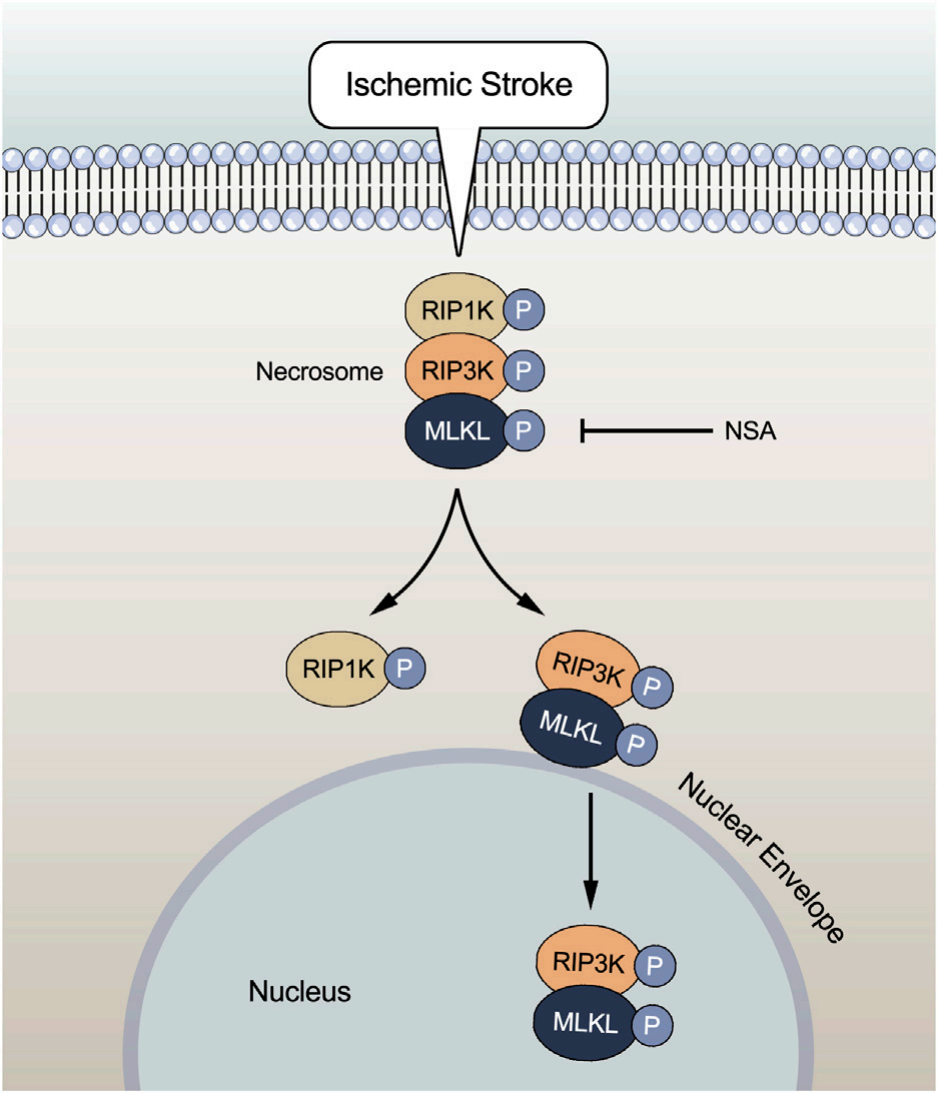
Ischemic stroke induces increases in protein levels of necroptotic key kinases such as MLKL/p-MLKL, RIP3K/p-RIP3K, and RIP1K/p-RIP1K. The complex of MLKL and RIP3K translocates to the nuclear envelope and nucleus, leading to necroptosis. NSA reduces the levels of these necroptotic kinases. Importantly, NSA blocks both the nucleus and nuclear envelope co-localization of p-MLKL and p-RIP3K induced by ischemic stroke, indicating that the translocation of p-MLKL, along with p-RIP3K, to the nuclear envelope and the nucleus may play a crucial role in MLKL-mediated necroptosis under ischemic conditions.

In summary, the current study provides important evidence that NSA exerts protective effects against focal ischemia/reperfusion injury through inhibiting necroptosis via preventing the upregulation of necroptosis kinases such as MLKL/p-MLKL, RIP3K/p-RIP3K, and RIP1K/p-RIP1K in ischemic penumbra of cerebral cortex and ischemic astrocytes, as well as blocking both the nucleus and nuclear envelope localization of p-MLKL and p-RIP3K. The translocation of p-MLKL, along with p-RIP3K, to the nuclear envelope and the nucleus may play a crucial role in MLKL-mediated necroptosis under ischemic conditions (Figure 7). How the nuclear complex of MLKL and RIP3K plays a role and whether the nuclear envelope co-localization of MLKL and RIP3K exerts independently at nuclear envelope in necroptosis after ischemic stroke remain to be investigated in the very near future.

## Data Availability

The original contributions presented in the study are included in the article/Supplementary Material, further inquiries can be directed to the corresponding authors.
