# Supplementary material for: The brain protection of MLKL inhibitor necrosulfonamide against focal ischemia/reperfusion injury associating with blocking the nucleus and nuclear envelope translocation of MLKL and RIP3K
Source: Front Pharmacol. 2023 Oct 24;14:1157054. doi: 10.3389/fphar.2023.1157054 (PMC10642205; doi:10.3389/fphar.2023.1157054)
Supplement: Supplementary file 1 [file DataSheet2.PDF]

Supplementary Material

The brain protection of MLKL inhibitor necrosulfonamide against focal ischemia/reperfusion injury associating with blocking the nucleus and nuclear envelope translocation of MLKL and RIP3K

Xian-Yong Zhou[†], Bo Lin[†], Wei Chen, Rui-Qi Cao, Yi Guo, Ali Said, Taous Khan, Hui-Ling Zhang[*], Yong-Ming Zhu[*]

*** Correspondence:**
Hui-Ling Zhang
zhanghuiling@suda.edu.cn;
huilingzhang07@hotmail.com

Yong-Ming Zhu
ymzhu@suda.edu.cn

†These authors have contributed equally to this work

Table 1 The antibodies for Western Blotting (WB) analysis

Protein	Antibody	Brand	Usage
RIP1K	#610458	BD	1:200
p-RIP1K	#31122	Cell Signaling Technology	1:1000
RIP3K	#NBP1-77299	Novus	1:1000
p-RIP3K	#ab195117	Abcam	1:1000
MLKL	#orb32399	Biorbyt	1:1000

p-MLKL	#ab196436	Abcam	1:1000
β-actin	#A5441	Sigma	1:5000
Lamin B1	#12978-1-AP	Proteintech	1:1000
GAPDH	#G8795	SIGMA	1:1000
Histone H3	#YT2166	immunoway	1:1000
Anti-mouse IgG (H + L)	#042-06-18-06	KPL	1:10,000
Anti-rabbit IgG (H + L)	#042-06-15-06	KPL	1:10,000
