# Supplementary material for: The brain protection of MLKL inhibitor necrosulfonamide against focal ischemia/reperfusion injury associating with blocking the nucleus and nuclear envelope translocation of MLKL and RIP3K
Source: Front Pharmacol. 2023 Oct 24;14:1157054. doi: 10.3389/fphar.2023.1157054 (PMC10642205; doi:10.3389/fphar.2023.1157054)
Supplement: Supplementary file 2 [file DataSheet1.pdf]

Original Data

Fig 1A (1/3)

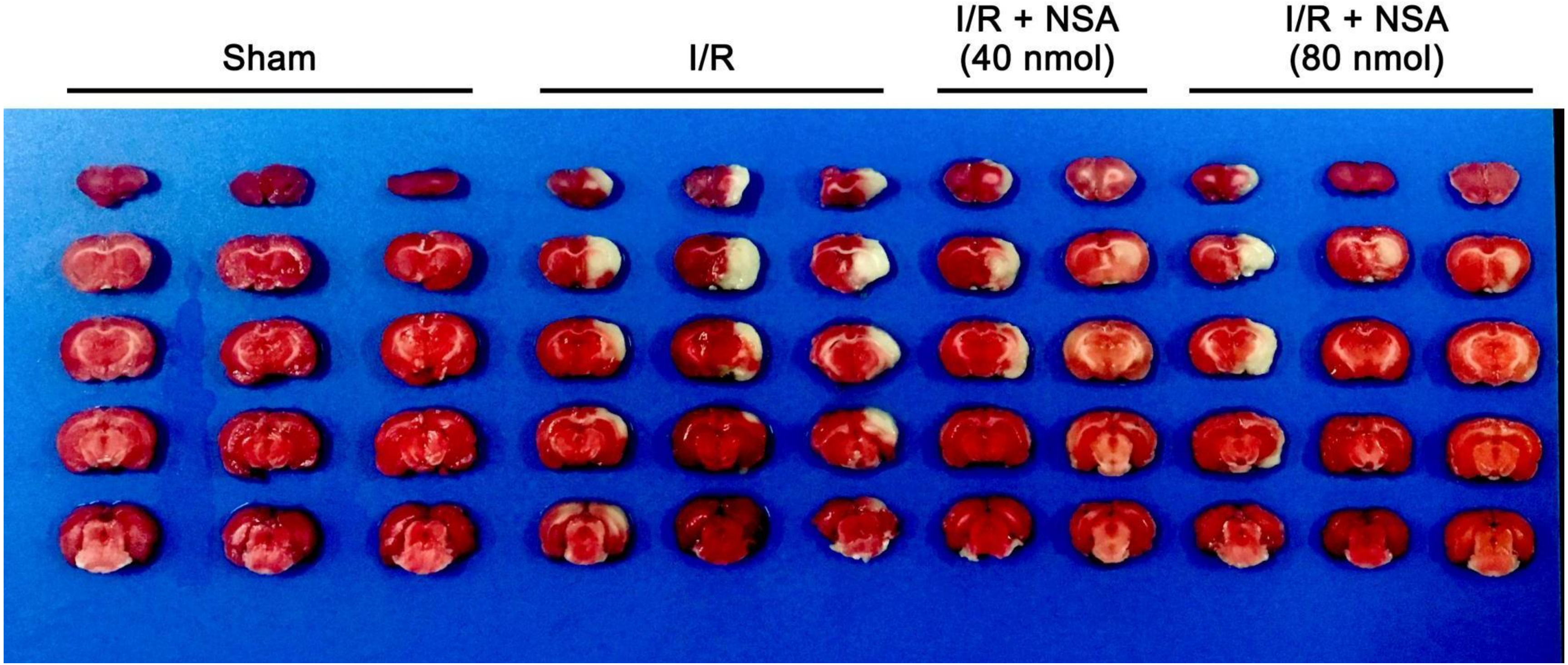

Fig 1A (2/3)

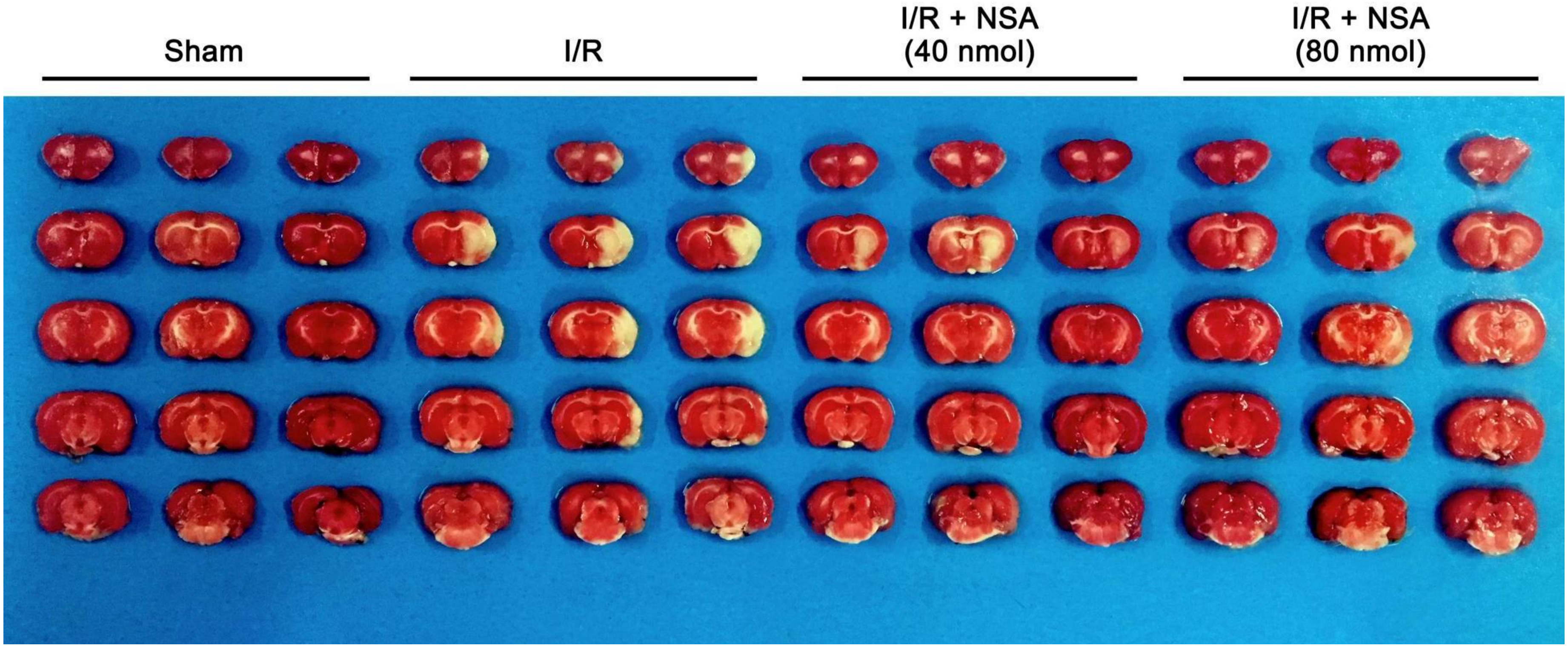

Fig 1A (3/3)

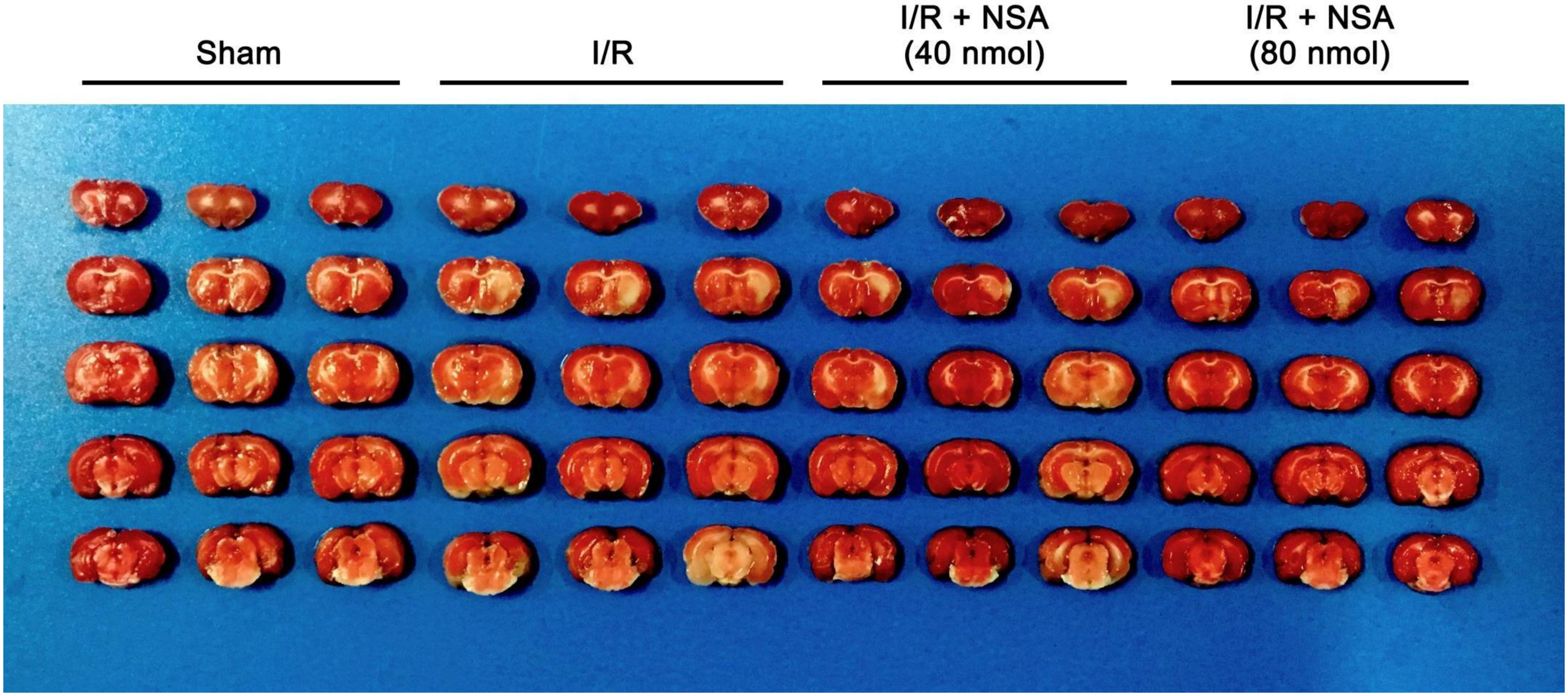

Fig 2A (1/2)

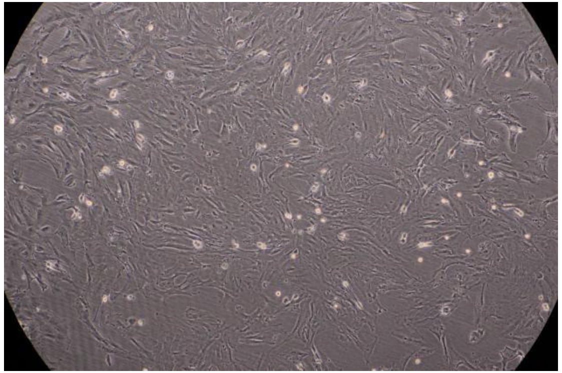

non-OGD/Re

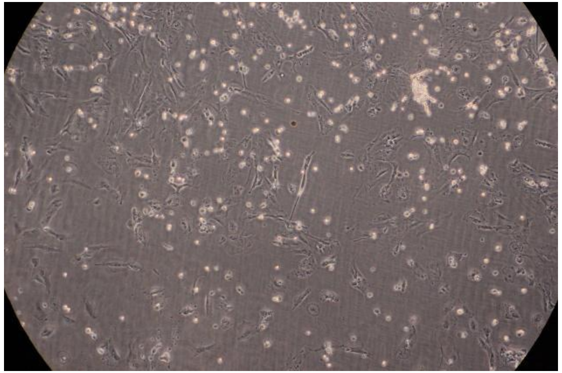

OGD/Re

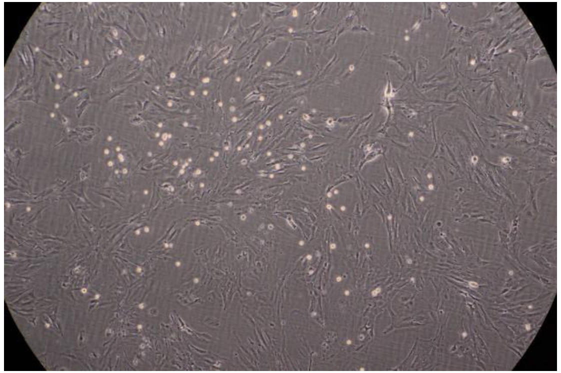

OGD/Re + 0.1 μM NSA

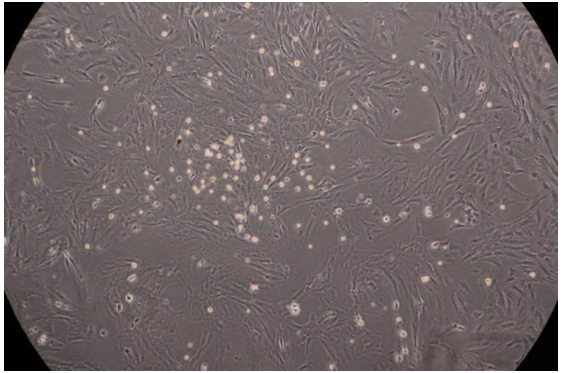

OGD/Re + 1 μM NSA

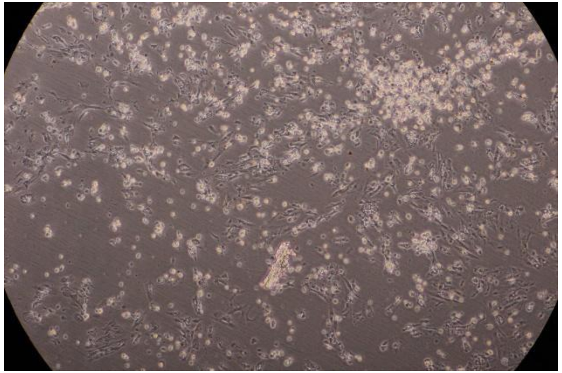

OGD/Re + 10 μM NSA

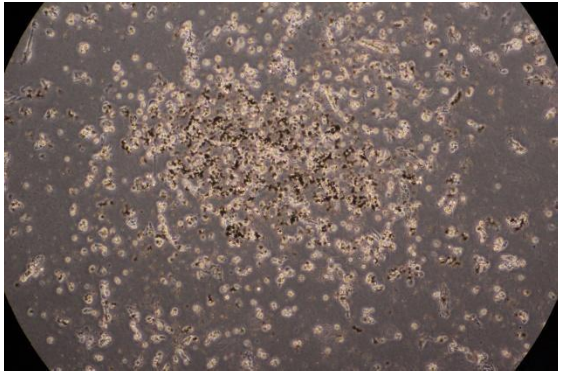

OGD/Re + 100 μM NSA

Fig 2A (2/2)

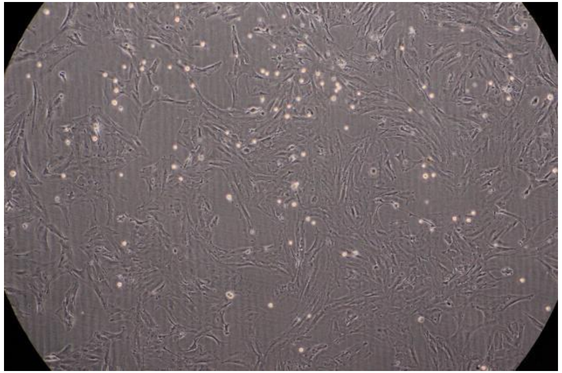

OGD/Re + Nec-1

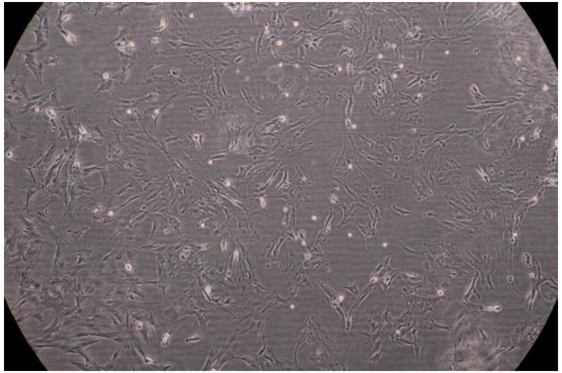

OGD/Re + GSK-872

Fig 2C (1/2)

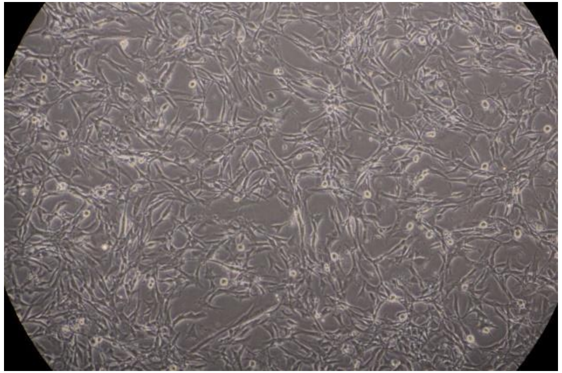

non-OGD

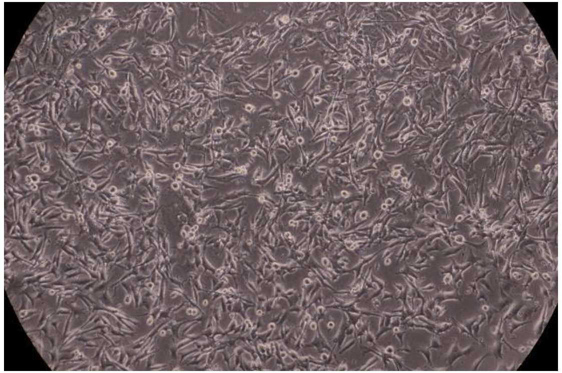

OGD/Re

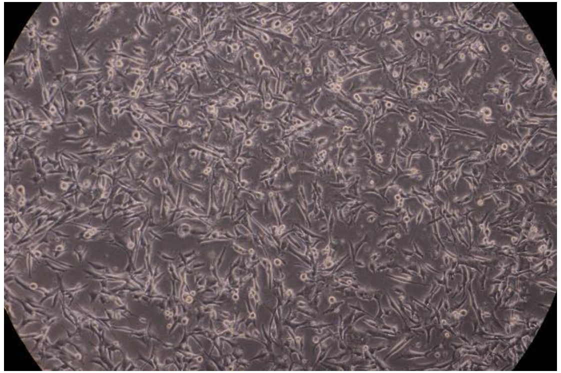

OGD/Re+0.1μM NSA

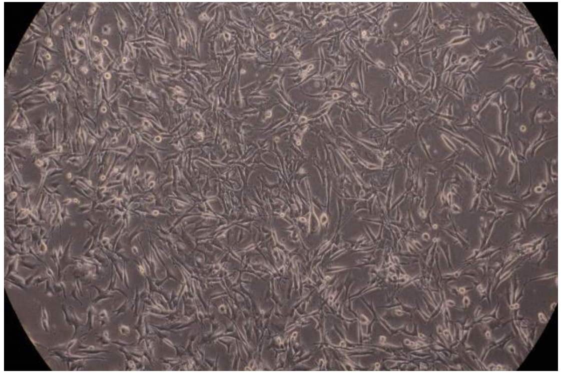

OGD/Re+1μM NSA

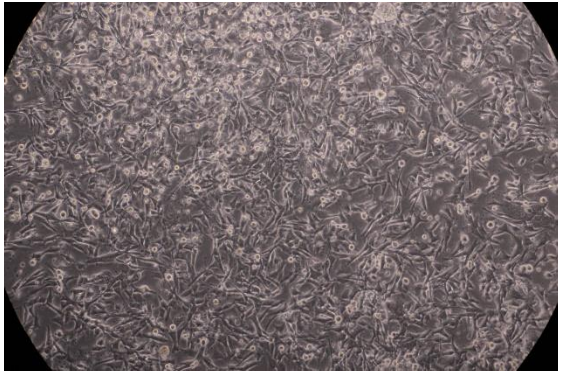

OGD/Re+10μM NSA

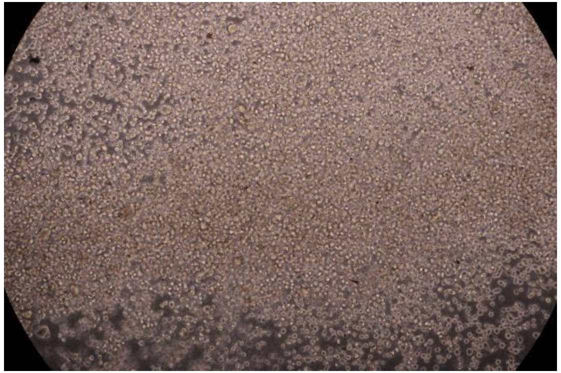

OGD/Re+100μM NSA

Fig 2C (2/2)

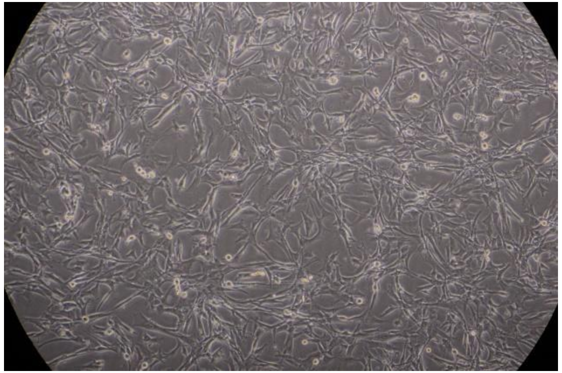

OGD/Re+Nec-1

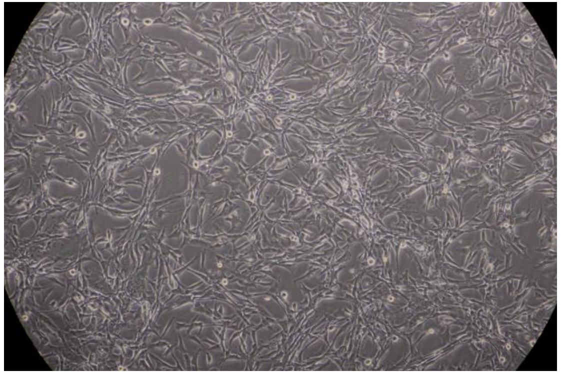

OGD/Re + GSK-872

Fig 3A

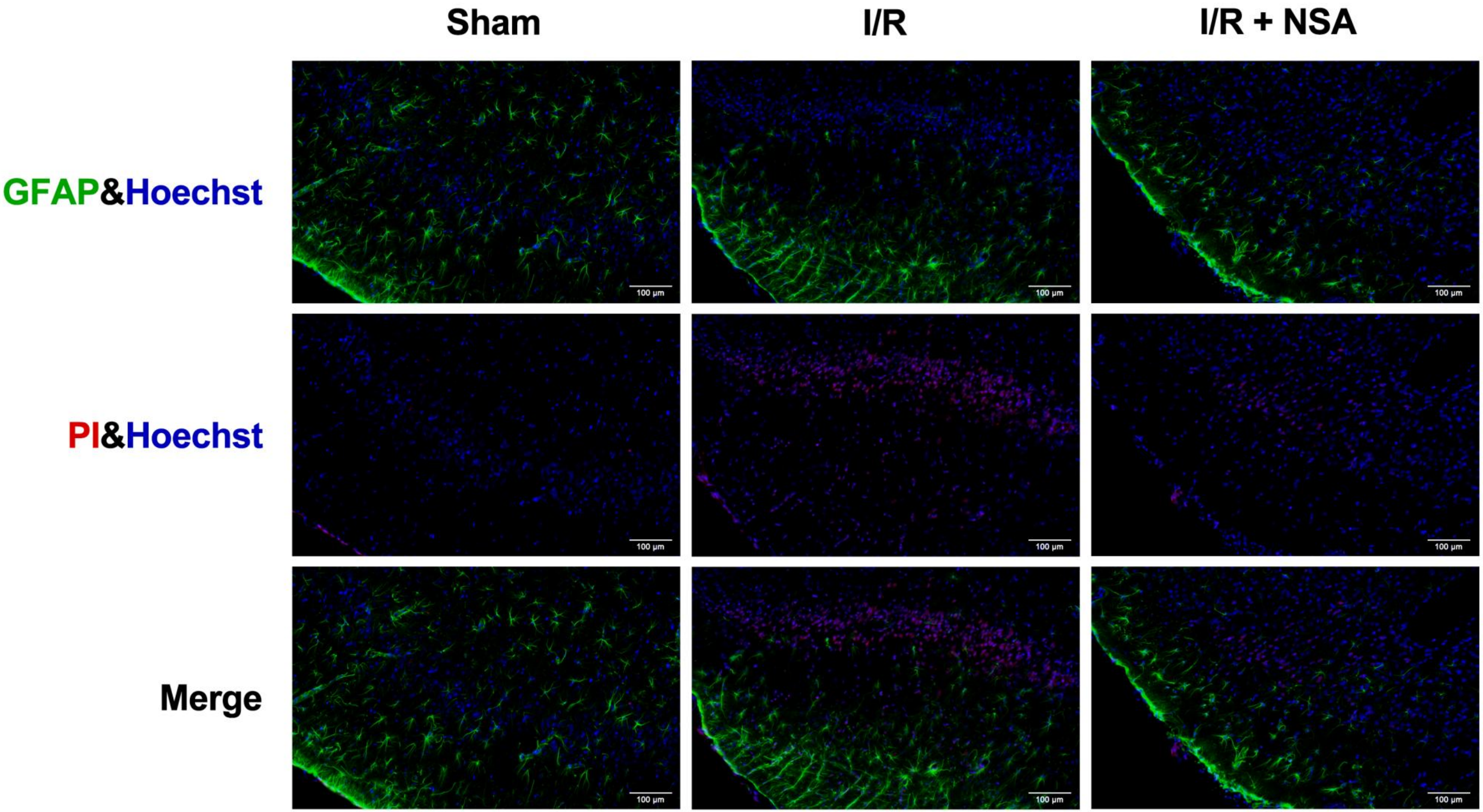

Fig 3C

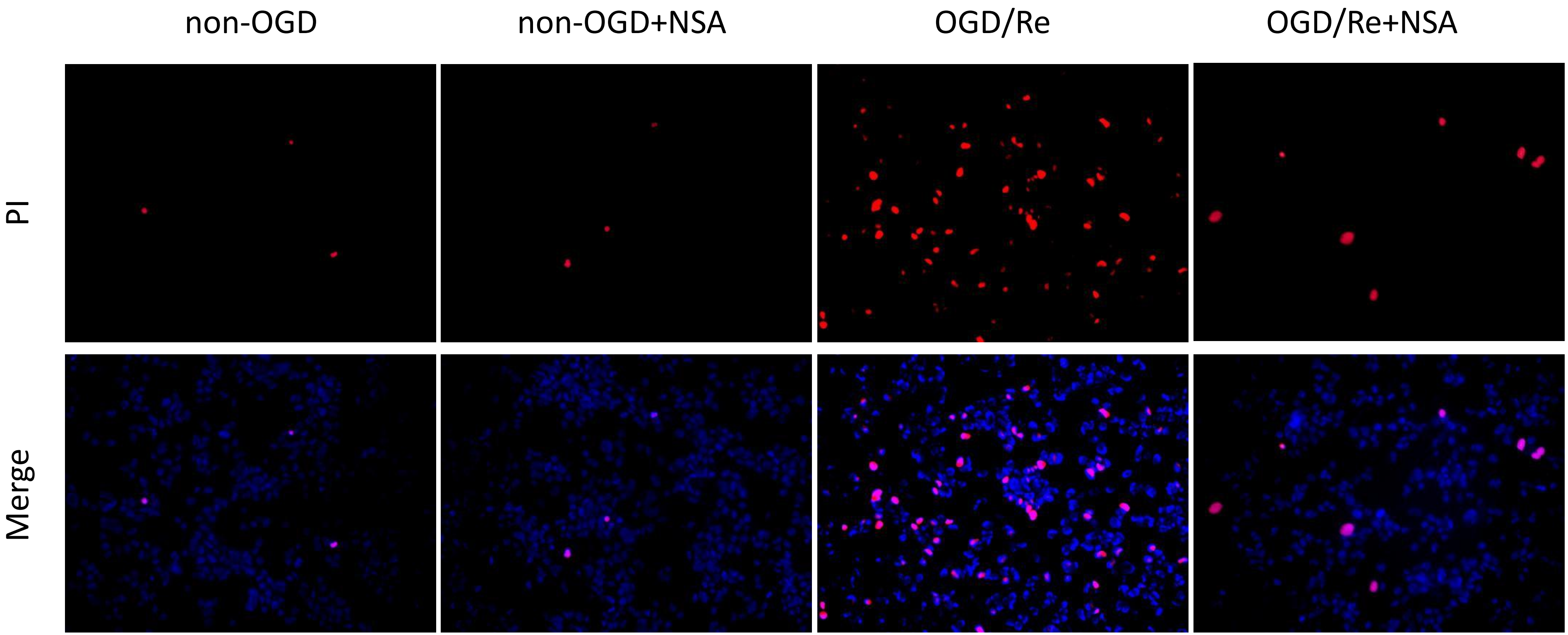

Fig 4A

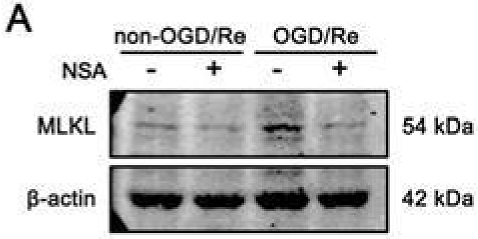

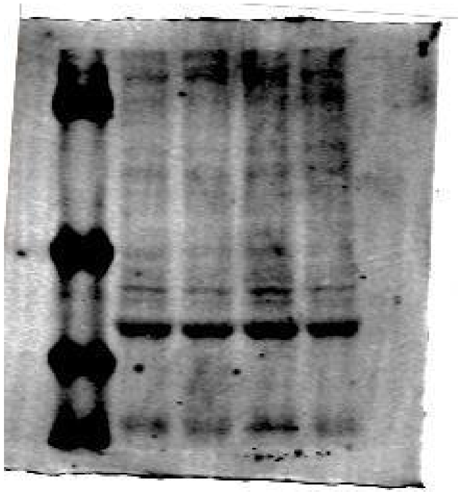

MLKL
β-actin

Fig 4B

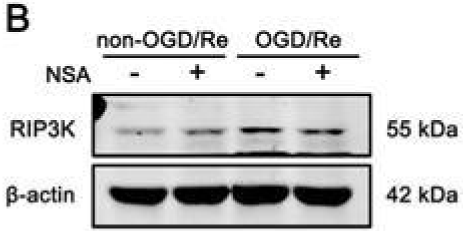

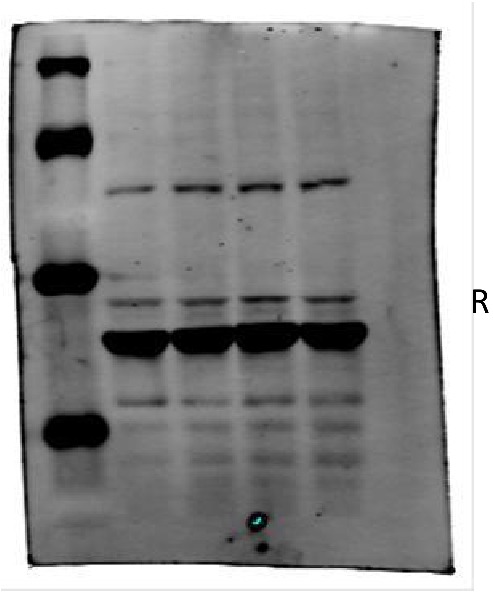

RIP3K

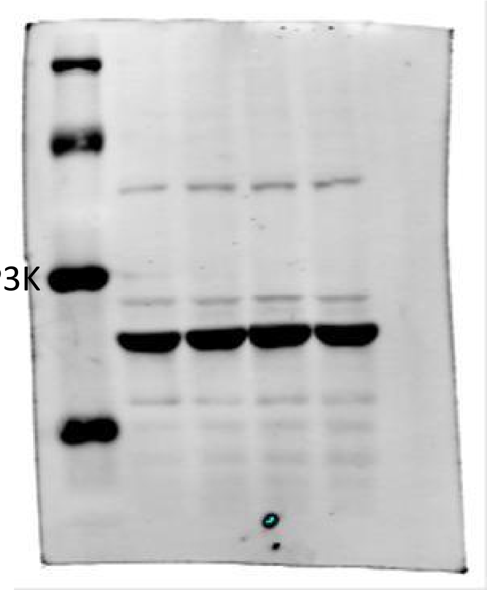

β-actin

Fig 4C

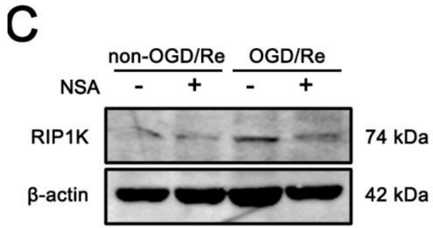

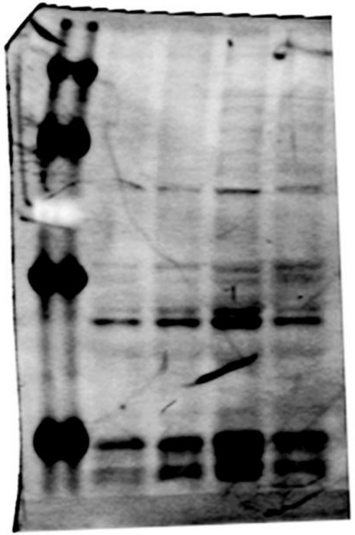

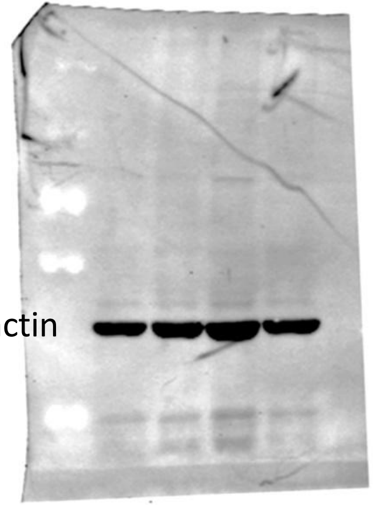

Fig 4G

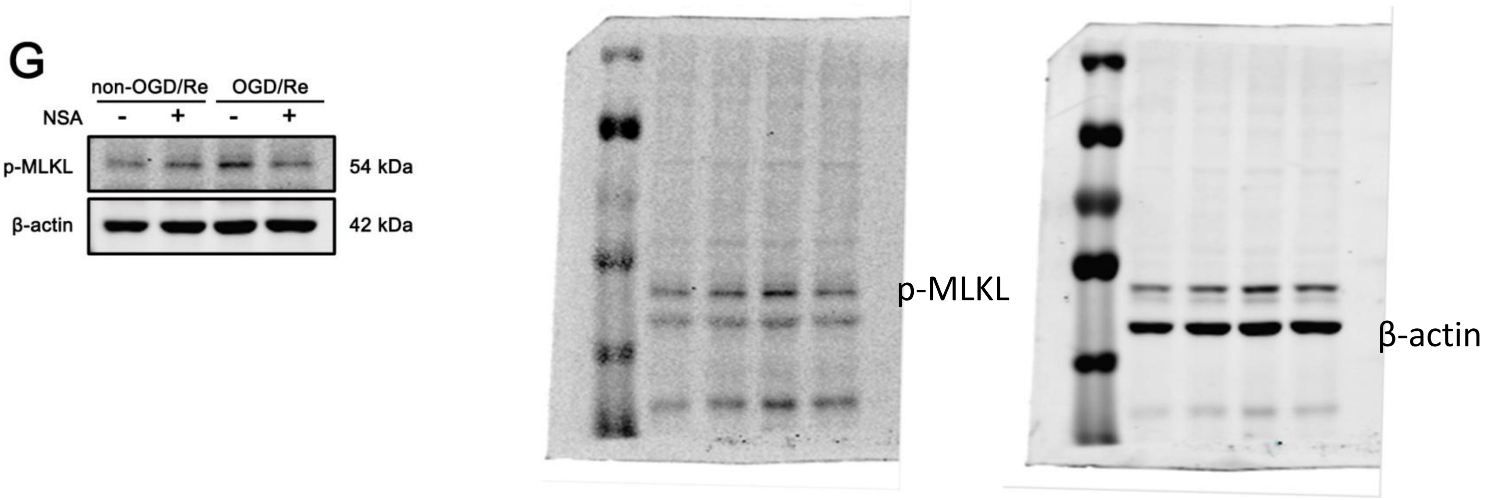

Fig 4H

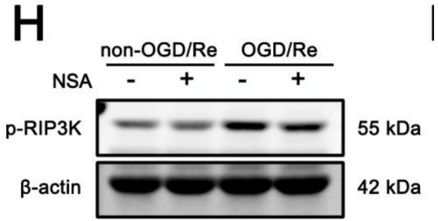
H
non-OGD/Re
OGD/Re
NSA
-
+
-
+
p-RIP3K
55 kDa
β-actin
42 kDa

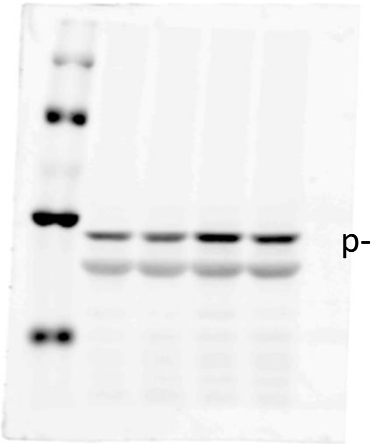
p-RIP3K

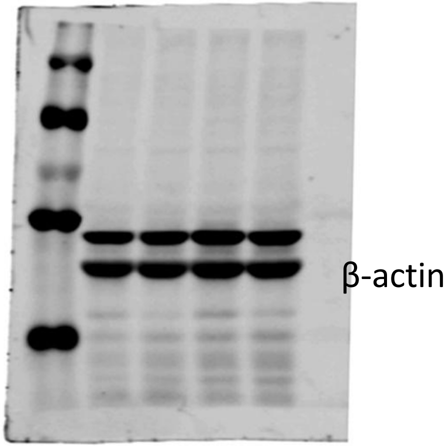
β-actin

Fig 4I

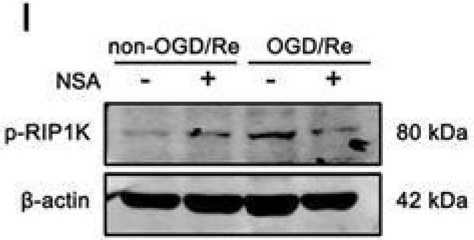

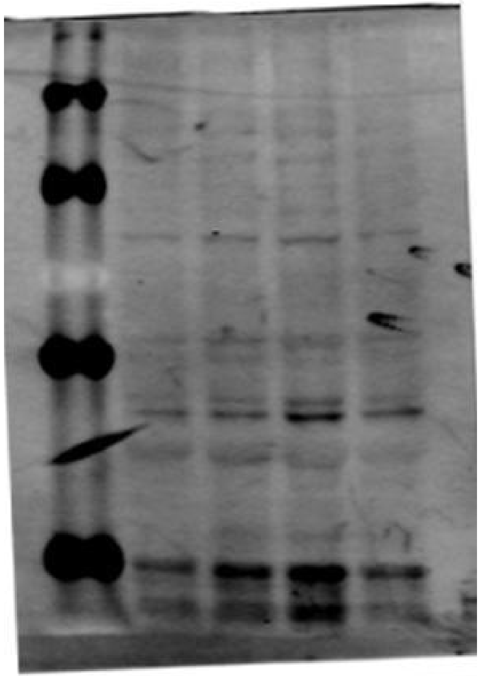

p-RIP1K

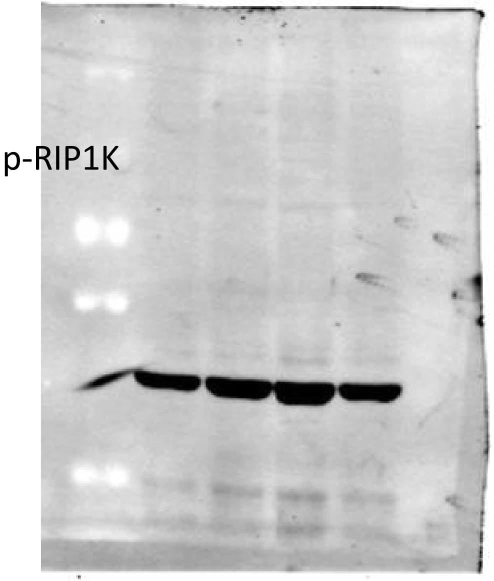

β-actin

Fig 5 (1/4)

A

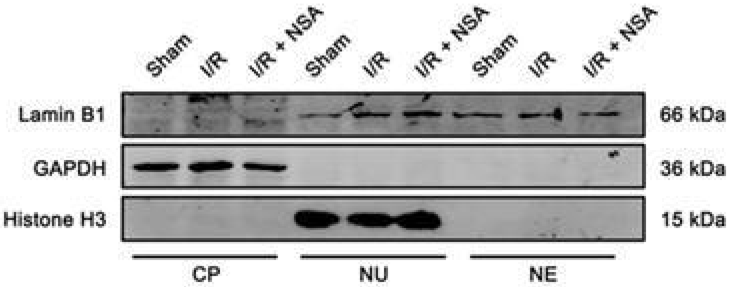
Sham
I/R
I/R + NSA
Sham
I/R
I/R + NSA
Sham
I/R
I/R + NSA
Lamin B1
GAPDH
Histone H3
66 kDa
36 kDa
15 kDa
CP
NU
NE

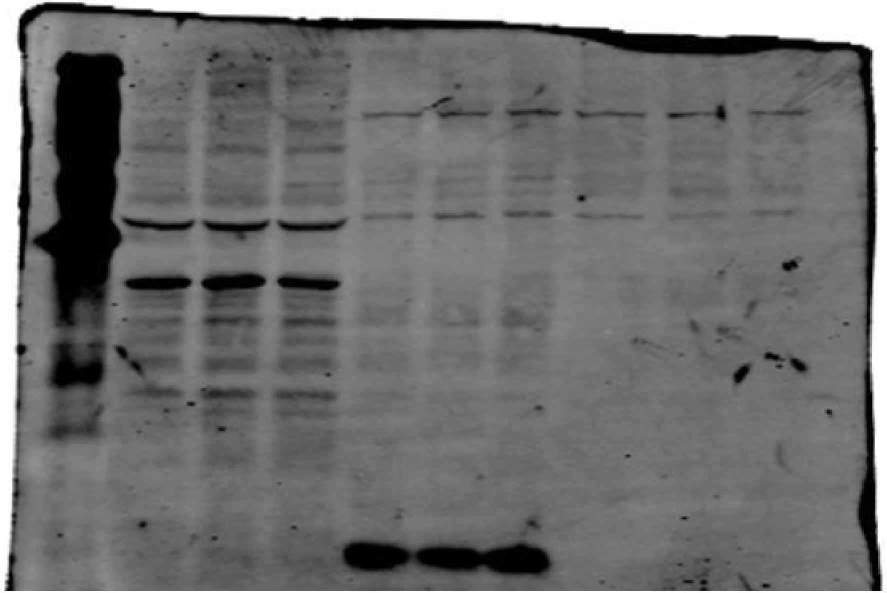
Lamin B1
GAPDH
H3

Fig 5 (2/4)

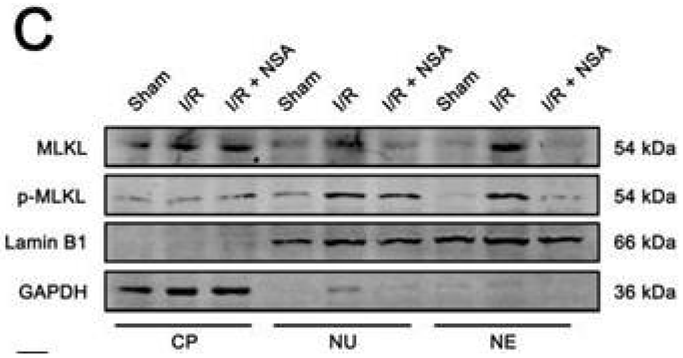

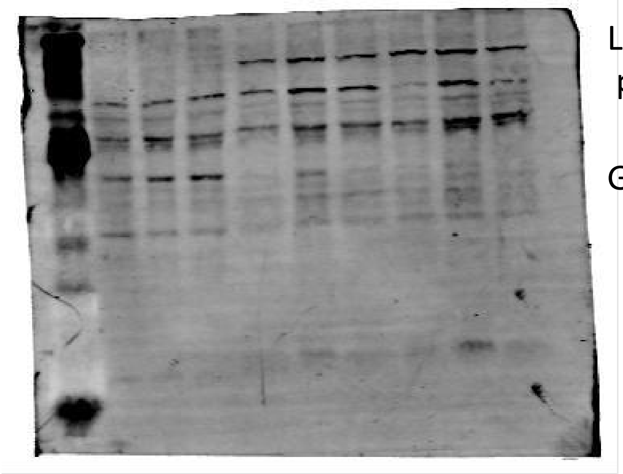

Lamin B1
p-MLKL
GAPDH

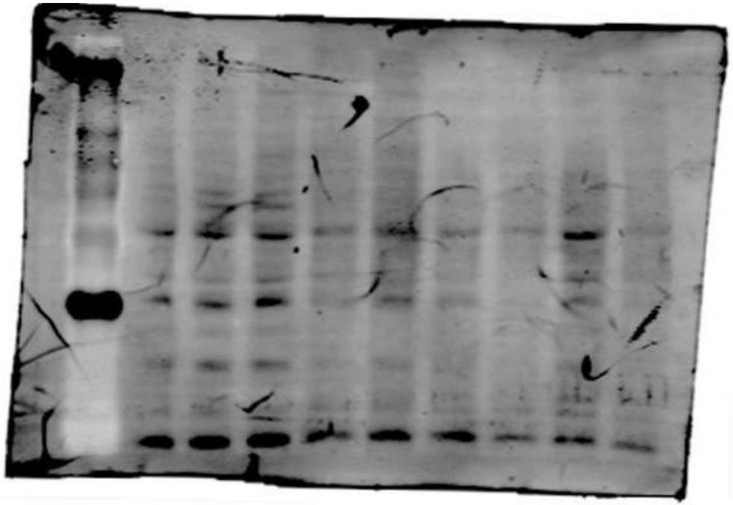

MLKL

Fig 5 (3/4)

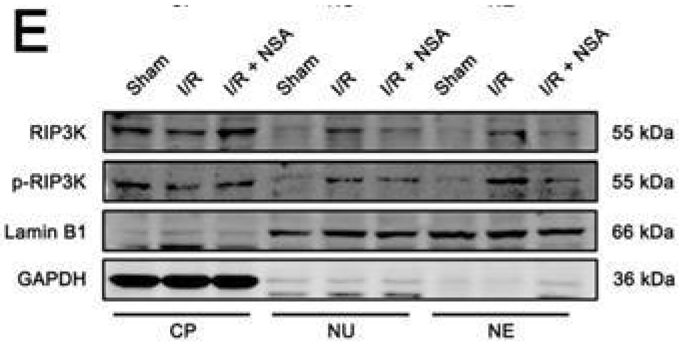

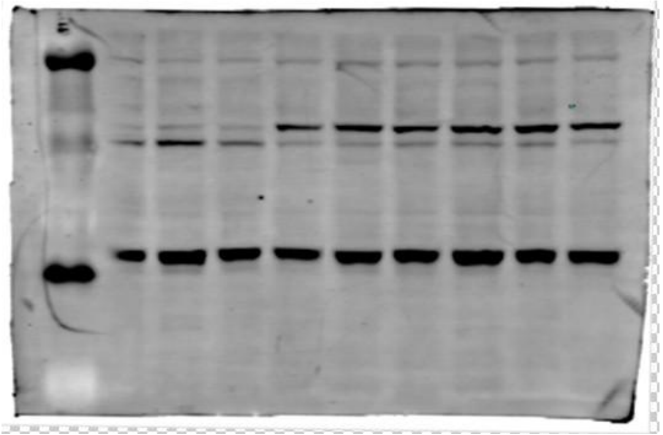

Lamin B1

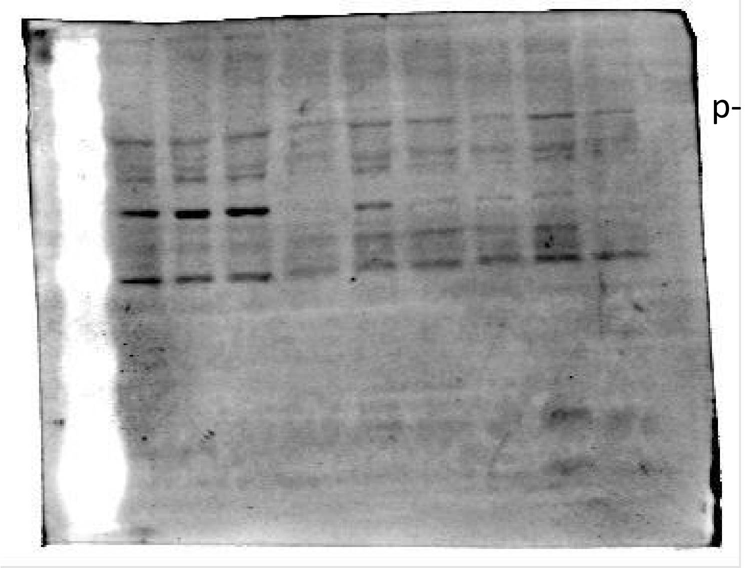

p-RIP3K

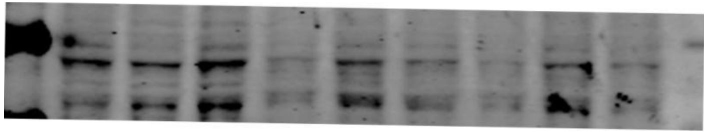

RIP3K

Fig 5 (4/4)

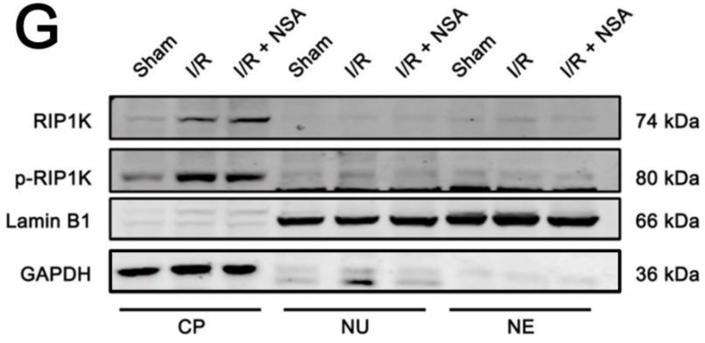
G
Sham
I/R
I/R + NSA
Sham
I/R
I/R + NSA
Sham
I/R
I/R + NSA
RIP1K
p-RIP1K
Lamin B1
GAPDH
74 kDa
80 kDa
66 kDa
36 kDa
CP
NU
NE

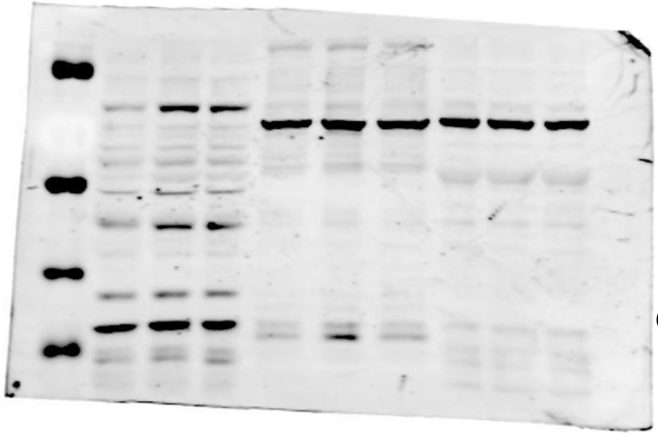
p-RIP1K
Lamin B1
GAPDH

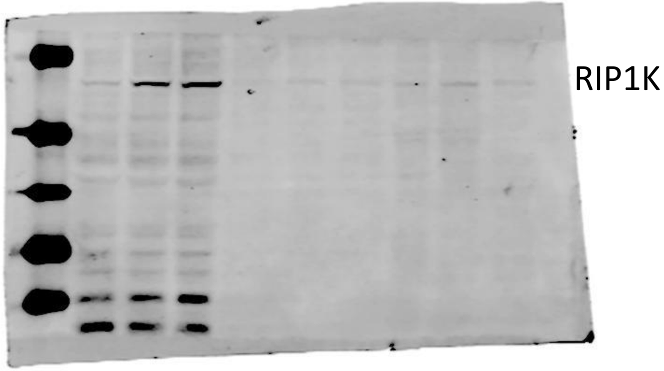
RIP1K

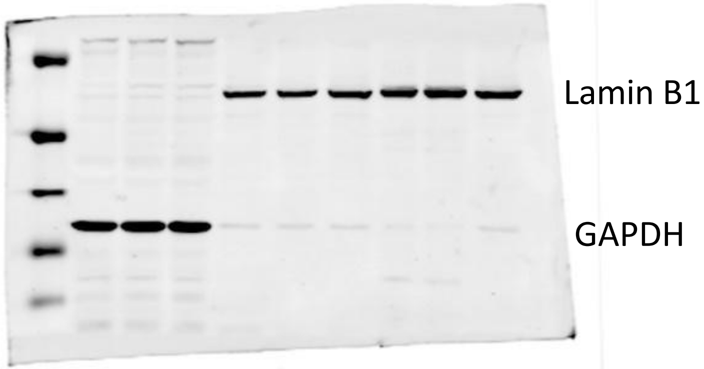
Lamin B1
GAPDH

Fig 6A

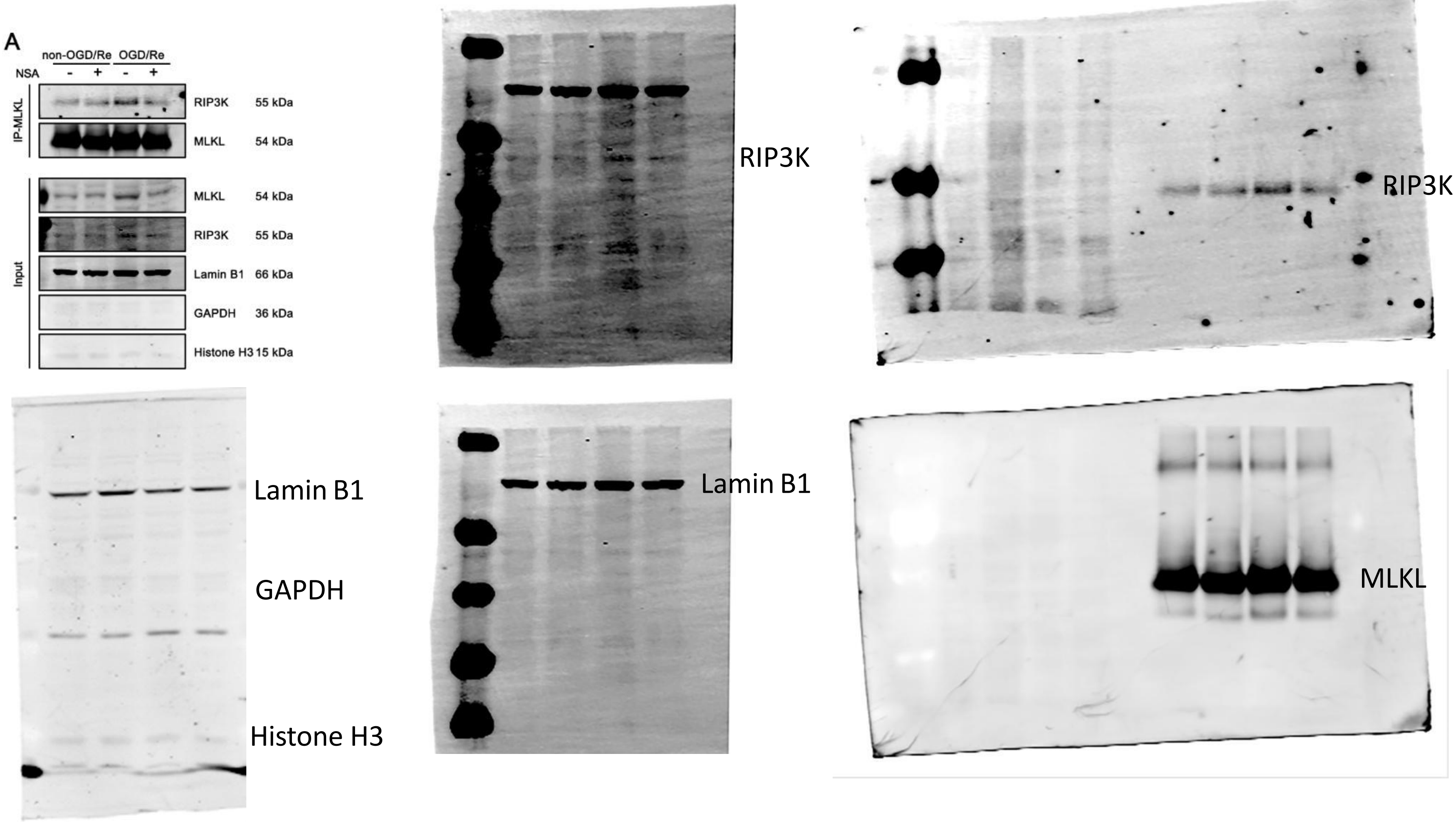
A
non-OGD/Re
OGD/Re
NSA - + - +
IP-MLKL
Input
RIP3K 55 kDa
MLKL 54 kDa
MLKL 54 kDa
RIP3K 55 kDa
Lamin B1 66 kDa
GAPDH 36 kDa
Histone H3 15 kDa
RIP3K
RIP3K
Lamin B1
GAPDH
Histone H3
Lamin B1
MLKL

Fig 6C

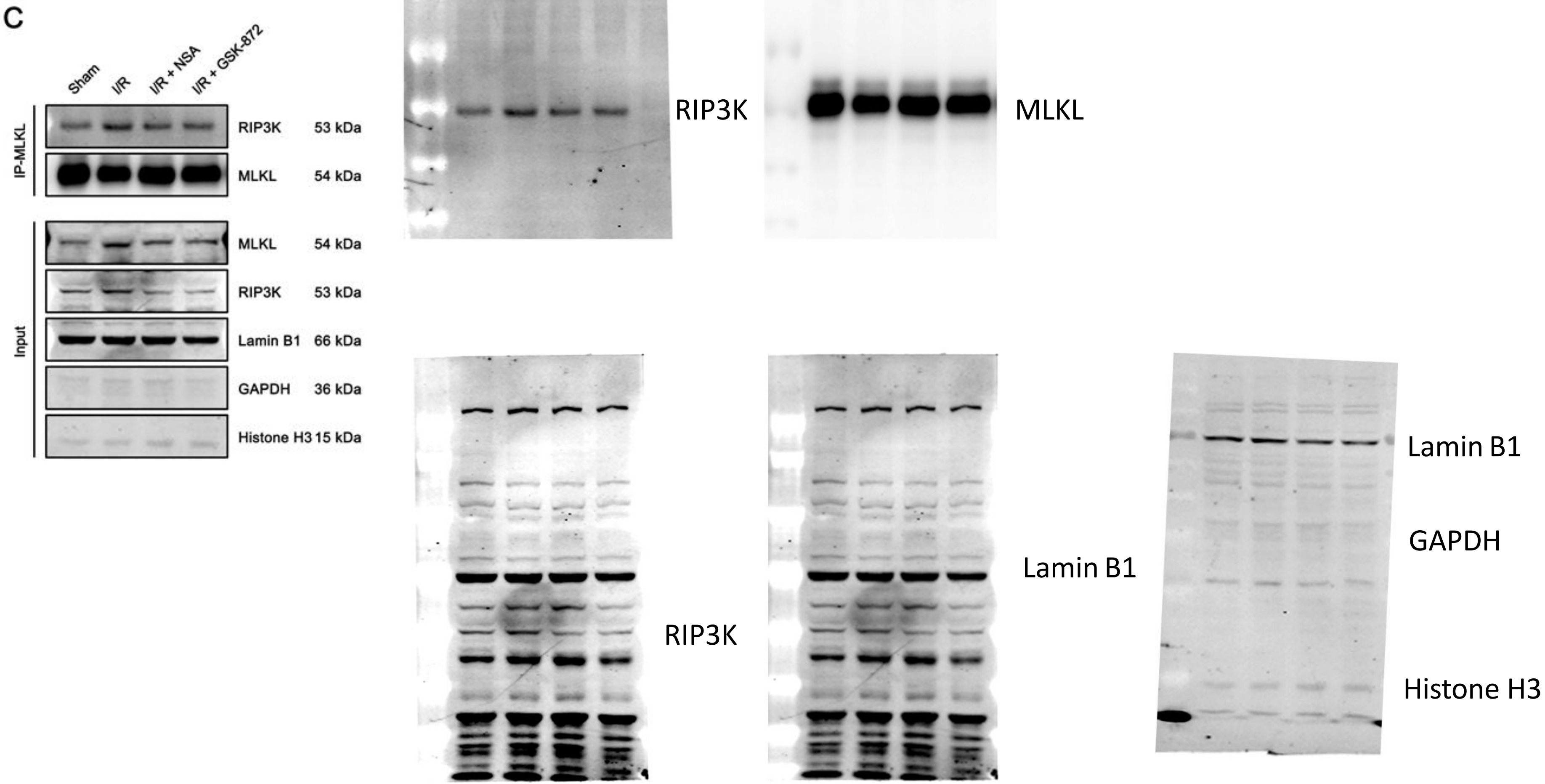
C
Sham
I/R
I/R + NSA
I/R + GSK-872
IP-MLKL
RIP3K 53 kDa
MLKL 54 kDa
Input
MLKL 54 kDa
RIP3K 53 kDa
Lamin B1 66 kDa
GAPDH 36 kDa
Histone H3 15 kDa
RIP3K
MLKL
RIP3K
Lamin B1
Lamin B1
GAPDH
Histone H3
